# Deletion of *Mir223* Exacerbates Lupus Nephritis by Targeting *S1pr1* in *Fas^lpr/lpr^* Mice

**DOI:** 10.3389/fimmu.2020.616141

**Published:** 2021-01-26

**Authors:** Sumie Hiramatsu-Asano, Katsue Sunahori-Watanabe, Sonia Zeggar, Eri Katsuyama, Tomoyuki Mukai, Yoshitaka Morita, Jun Wada

**Affiliations:** ^1^ Department of Nephrology, Rheumatology, Endocrinology and Metabolism, Graduate School of Medicine, Dentistry and Pharmaceutical Sciences, Okayama University, Okayama, Japan; ^2^Department of Rheumatology, Kawasaki Medical School, Kurashiki, Japan

**Keywords:** miR-223-3p, *S1pr1*, S1PR1^+^CD4^+^ T cells, lupus nephritis, MRL/MpJ-*Fas^lpr^*/J mice

## Abstract

**Objective:**

The micro RNAs (miRNAs) and their target mRNAs are differentially expressed in various immune-mediated cells. Here, we investigated the role of *Mir223* and sphingosine-1-phosphate receptor 1 (*S1pr1*) in the pathogenesis of systemic lupus erythematosus.

**Methods:**

We analyzed miRNA and mRNA profiling data of CD4^+^ splenic T cells derived from MRL/MpJ-*Fas^lpr^*/J mice. We performed 3′ untranslated region (UTR) luciferase reporter gene assay using human umbilical vein endothelial cells (HUVECs). We generated the B6-*Mir223*^−/−^*Fas^lpr/lpr^* mice and the lupus phenotypes were analyzed.

**Results:**

In CD4^+^ splenic T cells, we identified upregulation of miR-223-3p and downregulation of the possible target, *S1pr1* by RNA sequencing of MRL/MpJ-*Fas^lpr^*/J mice. The transfection with miR-223-3p mimic significantly suppressed a luciferase activity in HUVEC treated with a Lentivirus vector containing 3′ UTR of *S1pr1*. The mRNA levels of *S1pr1* were significantly decreased after miR-223-3p overexpression. In B6-*Mir223*^−/−^*Fas^lpr/lpr^* mice, the proportion of CD3^+^ T cells, CD3^+^CD4^-^CD8^−^ cells, B cells, plasma cells, and S1PR1^+^CD4^+^ T cells in the spleen was significantly increased compared with that in B6-*Mir223*^+/+^*Fas^lpr/lpr^* mice by flow cytometry. B6-*Mir223*^−/−^*Fas^lpr/lpr^* mice demonstrated the elevation of glomerular and renal vascular scores associated with enhanced intraglomerular infiltration of S1PR1^+^CD4^+^ T cells.

**Conclusion:**

Unexpectedly, the deletion of *Mir223* exacerbated the lupus phenotypes associated with increased population of S1PR1^+^CD4^+^ T in spleen and the enhanced infiltration of S1PR1^+^CD4^+^ T cells in inflamed kidney tissues, suggesting compensatory role of *Mir223* in the pathogenesis of lupus nephritis.

## Introduction

Systemic lupus erythematosus (SLE) is a chronic autoimmune disease associated with the overproduction of autoantibodies and infiltration of autoreactive B and T lymphocytes in lymphoid and non-lymphoid organs. Despite therapeutic advancements, the improvement of mortality or the development of end-stage renal disease has not been demonstrated and the development for new effective and targeted drugs is urgently required (1). Genetic, environmental, hormonal, epigenetic, and immunoregulatory factors are associated with the pathogenesis of SLE (2). DNA methylation, histone modification, and altered micro RNA (miRNA) are widely recognized as the key epigenetic mechanisms. miRNAs target specific mRNAs for mRNA stability and can fine-tune the expression of multiple mRNAs. In the patients with SLE and MRL/MpJ-*Fas^lpr^*/J (MRL/lpr) lupus-prone mice, several identified miRNAs affect the central pathway of SLE (3); for instance, upregulated miR-21 regulates lymphocyte signaling (4) and miR-155 deficiency which suppresses lupus activity by targeting sphingosine 1-phosphate (S1P) receptor 1 (*S1pr1*) (5). We previously performed global miRNA and mRNA profiling in CD4^+^ T cells purified from the spleen of MRL/lpr mice and compared with the C57BL6/J (B6). We identified miR-200a-3p and reported that it is involved in the hypoproduction of IL-2 in T cells by targeting CtBP2 complex in MRL/lpr mice (6). Therefore, the study of the role of miRNAs may provide important clues for potential future therapies in SLE patients.

In the present study, we investigated the roles of miR-223-3p and *S1pr1* mRNA in the MRL/lpr lupus-prone mice. *Mir223* is highly expressed and tightly regulated in hematopoietic cells, especially myeloid cells, for controlling excessive innate immune responses (7). *Mir223* in CD4^+^ T cells inhibit the human immunodeficiency virus (HIV) activity by targeting 3′ end of HIV genome (8). In autoimmune diseases, upregulation of *Mir223* in peripheral CD4^+^ T cells, especially Th17, has a potential role in progression of multiple sclerosis (9). In SLE patients, *Mir223* has been only explored as immunological biomarkers for disease pathophysiology. Although upregulation of *Mir223* in peripheral plasma was reported (10, 11), the expression of *Mir223* in peripheral plasma was significantly decreased in SLE patients with active nephritis (12).

*S1pr1* has been known to be expressed in several cell types of the immune system. Especially, S1PR1 on T cells plays a pivotal role in T cell circulation among secondary lymphoid organs dependent on S1P concentration (13–15). *S1pr1* conventional knockout mice exhibited intrauterine death by incomplete vascular maturation (16). T cell-specific *S1pr1* knockout mice showed a block in the egress of T cell from thymus into circulation and a reduction of lymphocytes, especially CD3^+^ cells, in secondary lymphoid organs (13). The S1P and S1PR1 signaling in T cells is also essential for T cell survival (17). The *S1pr1* expression of peripheral blood mononuclear cells was decreased in SLE patients and B6.MRL-*Fas^lpr^* mice (5). Agonists of S1PR1 suppressed the development of autoimmunity and renal injury by inducing sequestration of peripheral lymphocytes including autoreactive T cells into secondary lymphoid organs, reducing their infiltrates in target organs, and inducing its apoptosis in MRL/lpr mice (18–23). As mentioned above, miR-155 knockout lupus-prone B6.MRL-*Fas^lpr^* mice showed milder SLE clinical features than B6.MRL-*Fas^lpr^* mice by targeting *S1pr1* (5). Although the role of *S1pr1* in SLE is not completely understood, the miRNAs regulating the expression of *S1pr1* are expected critically involved in the pathogenesis of SLE. Here, we demonstrated that miR-223-3p plays a critical role in the regulation of T cell circulation and apoptosis by targeting S1PR1 in lupus prone mouse. The current investigation provides new data on the epigenetic control of T cell circulation in SLE.

## Materials and Methods

### Mice

B6.Cg-*Ptprc^a^Mir223^tm1Fcam^*/J (referred to as B6-*Mir223*^−^*^/^*^−^*Ptprc^a/a^*) on background of C57BL/6J (*Ptprc^a^*) (24), genetically lupus-prone female MRL/MpJ-*Fas^lpr^*/J (referred as MRL/lpr or MRL-*Fas^lpr/lpr^Ptprc^b/b^*), B6.MRL-*Fas^lpr^*/J (referred to as B6/lpr or B6-*Fas^lpr/lpr^Ptprc^b/b^*) on background of C57BL/6J (*Ptprc^b^*) (Jackson Laboratory), and C57BL/6J (*Ptprc^b^*) (referred as B6) (Charles River Laboratories) were purchased. The *Mir223* gene is located on the X chromosome. The animals were maintained in a 12-h light/dark cycle, with free access to water and standard rodent chow. The following animal experiments were approved by the Animal Care and Use Committee of the Department of Animal Resources, Advanced Science Research Center, Okayama University under the approval numbers of OKU-2013092, OKU-2015569, OKU-2015658, OKU-2015661, OKU-2015662, OKU-2015663, OKU-2015664, OKU-2016191, OKU-2016192, OKU-2016365, OKU-2016383, OKU-2016384, OKU-2016385, OKU-2017499, OKU-2017500, OKU-2017501, OKU-2017502, OKU-2018474, OKU-2018475, OKU-2018582, OKU-2018583, OKU-2018583, and OKU-2018585. All animal experiments were performed in accordance with relevant guidelines and regulations.

### mRNA and miRNA Expression Profiling by RNA Sequencing

Total RNA, including miRNA, was purified from CD4^+^ T cells of MRL/lpr mice and B6 mice using a miRNeasy mini kit (Qiagen). mRNA and miRNA sequencing and expression profile data were prepared as previously described (6). All raw and processed data are freely accessible in the Gene Expression Omnibus (https://www.ncbi.nlm.nih.gov/geo/) under the accession number GSE87219.

### 3′ Untranslated Region Luciferase Reporter Gene Assay

Human Umbilical Vein Endothelial Cells (HUVECs; CC-2519, Lonza) were cultured in EGM-2 medium (CC-3162, Lonza) supplemented with 10% fetal bovine serum (FBS) in a humidified atmosphere containing 5% CO_2_ at 37°C. The culture medium was replaced every 1–2 days, cells at 85–90% confluence were passaged at a ratio of 1:3 confluence. The cells were used between passages two and five in the following experiments. MISSION 3′UTR Lenti GoClone containing 3′UTR of *S1PR1* (HUTR09173; S1PR1-3′UTR) and MISSION 3′UTR Lenti GoClone-Controls (HUTR001C-004C; Controls) were purchased from Sigma-Aldrich. The Lentivirus particles and 8 μg/ml Hexadimethrine bromide^®^ (H9268, Sigma-Aldrich) were added to HUVECs for 10 h and then replaced with fresh medium. After 24 h, the infected cells were subjected to selection with 0.25 μg/ml puromycin. The selected cells were further co-transfected with miR-223-3p mimic and its negative control (nontargeting miRNA) into HUVECs using RNAiMAX (Invitrogen). The cells were harvested after 24 h of transfection, and Renilla luciferase activity was measured using the Luciferase Reporter Assay System Kit (MLS0001, Sigma-Aldrich) and GloMax^®^ 20/20 Luminometer (Promega).

### Evaluation of Active and Chronic Lesions of Lupus Nephritis

For histology, mouse kidneys were fixed in 10% formalin for 24 h at 4°C, and 4 μm paraffin sections were stained with hematoxylin and eosin, periodic acid-Schiff (PAS) stain. Glomerular hypercellularity was evaluated by counting the number of nuclei per glomerular cross-section in 10 randomly selected glomerular cross-sections per mouse. The glomerular lesions were graded on a scale of 0–3 as previously described (25). The glomerular lesion index was calculated from the sum of the scores for 40 randomly selected glomerular cross-sections per mouse. Renal vascular lesions were graded on a scale of 0–3 as previously described (25). The vascular lesion index was calculated from the sum of the scores for all vessels per section. Tubulointerstitial lesions were graded on a scale of 0–4 as previously described (26). The tubulointerstitial lesion index was calculated from the sum of the scores for 30 consecutive high-power fields (HPF) at a magnification of ×400 in the cortex per section.

### Immunofluorescence

Mouse kidneys were immediately frozen at −80°C in OCT compound (Sakura, Japan), and 4 μm cryostat sections were stained with fluorescein isothiocyanate (FITC) conjugated goat anti-mouse IgG or rabbit anti-mouse C3 (Cappel). Staining of all sections was visualized with a fluorescence microscope (BX51; Olympus). Immunofluorescence intensity (measured as the number of pixels/µm^2^) was quantified using cellSens software (version 1.16; Olympus). At least five glomeruli per section were analyzed.

For immunofluorescence with CD4, CD8, and S1PR1, mouse kidneys were embedded in OCT compound (Sakura, Japan), and 4 μm cryostat sections were fixed in 4% paraformaldehyde (PFA). After blocking in 5% BSA, the sections were incubated with the appropriate primary antibodies; rat monoclonal anti-CD4 (1:50; RM4-5, 100505), rat monoclonal anti-CD8a (1:50; 53-6.7, 100701) (BioLegend), and rabbit polyclonal S1PR1(1:100; ab137467, Abcam). After overnight incubation, the sections were further treated with Alexa Fluor^®^ 647 conjugated goat anti-rat secondary antibody (ab150167, Abcam) or Alexa Fluor^®^ 594 conjugated goat anti-rabbit secondary antibody (ab150084, Abcam). Nuclear staining was performed using 4′,6-diamidino-2-phenylindole (DAPI; 422801, BioLegend). Staining of all sections was visualized with a fluorescence microscope (BZ-X700; Keyence). Quantitation of staining was graded based on the number of positive cells per glomerulus or per HPF for tubular infiltrates, with a minimum of 10 glomeruli of HPF per mouse examined.

### RNA Isolation and Real-Time RT-PCR

Total cellular RNAs from human and mouse samples were extracted with an RNeasy mini kit (Qiagen). cDNAs were reverse transcribed from mRNAs with a high-capacity cDNA RT kit (Thermo Fisher Scientific), while cDNAs from miRNAs with a TaqMan miRNA reverse transcription kit (Thermo Fisher Scientific). Real-time PCRs for miR-223-3p (002295), sno-202 (001232), sno-234 (001234) and RNU48 (001006) were performed using TaqMan primer/probes with TaqMan miRNA assays (Thermo Fisher Scientific). The expression of miRNAs was normalized to sno-202 and sno-234 for mice and RNU48 for human samples by the ΔΔCt method. Real-time PCRs for *S1pr1* (Mm02619656_s1), *Gapdh* (Mm99999915_g1), *Cxcl9* (Mm00434946_m1), *Cxcl10* (Mm00445235_m1), *Cxcl11* (Mm00444662_m1), *Ccl2* (Mm00441242_m1), *Ccl4* (Mm00443111_m1), *Ccl5* (Mm01302427_m1), *S1PR1* (Hs01922614_s1) and *GAPDH* (Hs02786624_g1) were performed using ABI TaqMan gene expression assays (Applied Biosystems) according to the manufacturer’s protocol and normalized to *GAPDH* by the ΔΔCt method.

### Systemic Lupus Erythematosus Patients and CD4^+^ T Lymphocyte Purification

The 15 SLE patients (10 females and five males) who fulfilled at least four of the 11 revised criteria of the American College of Rheumatology for the classification of SLE and 6 healthy controls were enrolled and peripheral CD4^+^ T lymphocytes were purified as previously described (27). The studies were approved by the Ethical Committee, Okayama University Hospital (#1779) and the written informed consent was obtained.

### Statistical Analyses

All results are shown as the mean ± standard error (SE) of data from at least three separate experiments, each performed with more than triplicate samples. Normal distribution of the data was assessed by Shapiro–Wilk test, and statistically significant differences between groups were determined using the Student’s 2-tailed *t*-test or Wilcoxon signed-rank test, as appropriate. The data were also analyzed with one-way analysis of variance and Tukey’s honestly significant difference test when multiple comparisons against the control were required. Pearson’s χ2 test was used to compare the distribution of glomerular lesions with grading scores from 0 to 3 (25), renal vascular lesions with grading scores from 0 to 3 (25) and tublointerstitial injury with grading scores from 0 to 4 (26). P values less than 0.05 were considered significant. All statistical analyses were performed using the JMP 11.2.0 software package (SAS Institute).

## Results

### Upregulated miR-223-3p and Repressed S1pr1 mRNA Levels in CD4^+^ T Cells of *MRL/lpr* Mice

**Figure 1:**
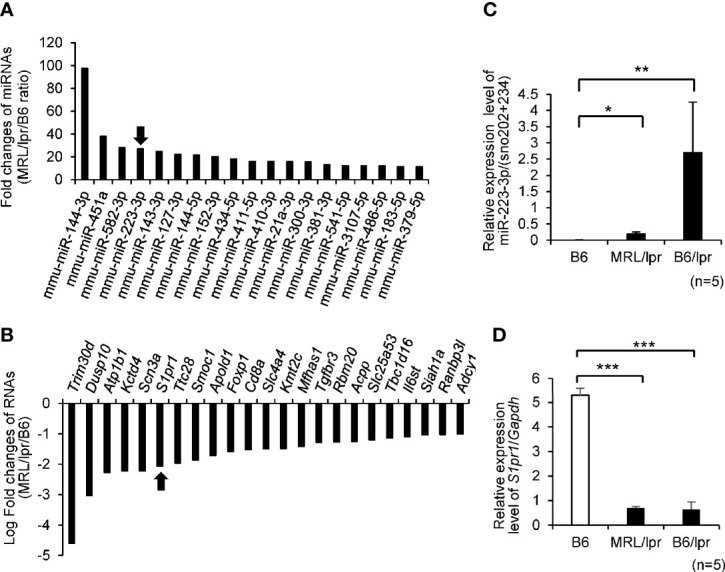
Top 20 differential expression levels of miRNAs and mRNAs in CD4+T cells isolated from MRL/lpr lupus-prone (MRL/lpr) *vs* C57BL/6 (B6) mice by microarray analysis. **(A)** Fold change (ratio between MRL/lpr/B6) in miRNA expression MRL/lpr *vs* B6. We focused on mmu-miR-223-3p which was indicated by arrow. **(B)** Candidate target genes for upregulated mmu-miR-223-3p were identified using the commonly used prediction algorithm, miRDB. These targets and our mRNA expression profiles were integrated. Fold change (ratio between MRL/lpr/B6) in mRNA expression MRL/lpr *vs* B6. We focused on *S1pr1* which was indicated by arrow. **(C)** The expression level of miR-223-3p was evaluated by TaqMan quantitative PCR in MRL/lpr mice and B6/lpr lupus-prone (B6/lpr) mice compared with B6 mice (n = 5 per group, 16-week-old female). **(D)** The expression of *S1pr1* in CD4^+^T cells of mice spleen was evaluated by TaqMan quantitative PCR (n =5 per group, 16-week-old female). Data are presented as mean ± SEM. *p < 0.05, **p < 0.01, ***p < 0.001, by Student’s t-test.

To identify new candidate miRNAs and their target mRNAs involved in the pathogenesis of SLE, we integrated miRNA and mRNA sequencing data in splenic CD4^+^ T cells isolated from MRL/lpr and B6 mice (GSE87219). A total of 19 miRNAs were upregulated in MRL/lpr compared with B6 mice with a cut-off value of >10-fold (**Figure 1A** and **Supplementary Table 1**). We further screened the sets of upregulated miRNA and >2-fold downregulated mRNAs, which were retrieved from miRDB (http://mirdb.org.) as predicted targets. Among them, we identified that upregulation of miR-223-3p was associated with downregulation of 23 mRNAs by RNA sequencing data (**Figure 1B** **and** **Supplementary Table 2**). We further investigated the downregulation of predicted (*S1pr1*) target mRNAs in MRL/lpr, B6/lpr and B6 mice. By quantitative real-time PCR, miR-223-3p was upregulated and *S1pr1* was most significantly downregulated in MRL/lpr and B6/lpr mice compared with B6 mice (**Figures 1C, D**). Although the mRNA levels of *S1pr1* were significantly decreased in splenic CD4^+^ T cells from MRL/lpr compared with those from B6, the protein levels of S1PR1 were rather increased in splenic CD4^+^ T cells from MRL/lpr compared with those from B6 (**Supplementary Figure 1A**). S1PR1 protein expression on surface of CD4^+^ T cells is known to be downregulated in the blood and upregulated in lymphoid organ (28). It was reported that S1P induces S1PR1 internalization *via* endosomal pathway (29), and it subsequently undergoes ubiquitylation and proteasomal degradation by the ubiquitin ligase WW domain containing E3 ubiquitin protein ligase 2 (Wwp2) (30, 31). The S1PR1 protein was supposed to be regulated by posttranslational modification in MRL/lpr mice and we further examined ubiquitination of S1PR1 protein in splenic CD4^+^ T cells from MRL/lpr and B6. The starting materials (SMs) demonstrated higher expression of S1PR1 protein in CD4^+^ T cells from MRL/lpr compared with B6, while it was barely detected in the eluted fractions (ELs) of highly purified ubiquitinated proteins by UbiQapture-Q Kit (**Supplementary Figure 1B**). Taken together, upregulation of S1PR1 protein in splenic CD4^+^ T cells from MRL/lpr was mediated by reduced ubiquitination of the S1PR1, although B6 was not an appropriate control for MRL/lpr.

**Figure 2:**
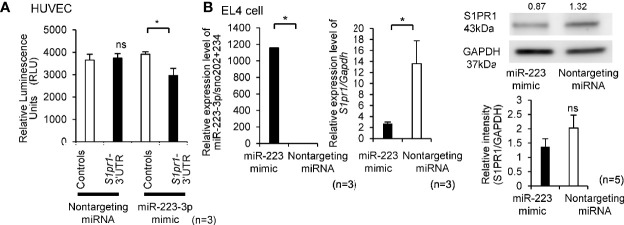
The miR-223-3p is a negative regulator for *S1pr1*. **(A)** HUVECs were co-transfected with nontargeting miRNA and miR-223-3p mimic with the no UTR or 3′UTR. Luciferase activity was assayed 24 h after transient co-transfection. Renilla luciferase activity was measured. (n = 3 per group). Data are presented as mean ± SEM. *p < 0.05, ns, not significant, by Wilcoxon signed-rank test. **(B)** The mRNA and protein level of *S1pr1* in EL4 cells was analyzed by TaqMan quantitative PCR and Western blot analyses after transfection with miR-223-3p mimic or nontargeting miRNA. The transfection efficacy of miR-223-3p mimic was confirmed by TaqMan quantitative PCR. miR-223-3p was transfected by lipofection, and the expression was compared with the nontargeting miRNA after 24 h transfection. The mRNA and protein levels of *S1pr1* after the overexpression of miR-223-3p was confirmed by TaqMan quantitative PCR and Western blot analyses. Numbers above the Western blots indicate band intensity (normalized to total GAPDH) measured by using ImageJ software. Quantification of Western blot results (right side). (n = 3 per group). Data are presented as mean ± SEM. *p < 0.05, ns, not significant, by Wilcoxon signed-rank test.

As shown in **Supplementary Table 3**, the 3′UTR region of *S1pr1* was predicted to serve as binding site for miR-223-3p in humans and mice. To determine whether *S1pr1* was a direct target of miR-223-3p, Lentivirus of S1pr1-3′UTR and Controls were infected to HUVECs for luciferase miRNA target assays. Co-transfection of miR-223-3p mimic demonstrated a significant reduction in luciferase activity, while nontargeting miRNA did not alter the luciferase activity (**Figure 2A**). To further confirm that *S1pr1* is a target of miR-223-3p, we detected the endogenous *S1pr1* mRNA and protein level after transiently transfecting mimic miR-223-3p into EL4 cells. As shown in **Figure 2B**, the expression level of *S1pr1* mRNA and protein decreased after miR-223-3p overexpression. Taken together, the miR-223-3p is a negative regulator for *S1pr1*.

### Reduction of S1PR1 mRNA in CD4^+^ T Cells From the Patients With Systemic Lupus Erythematosus

**Figure 3:**
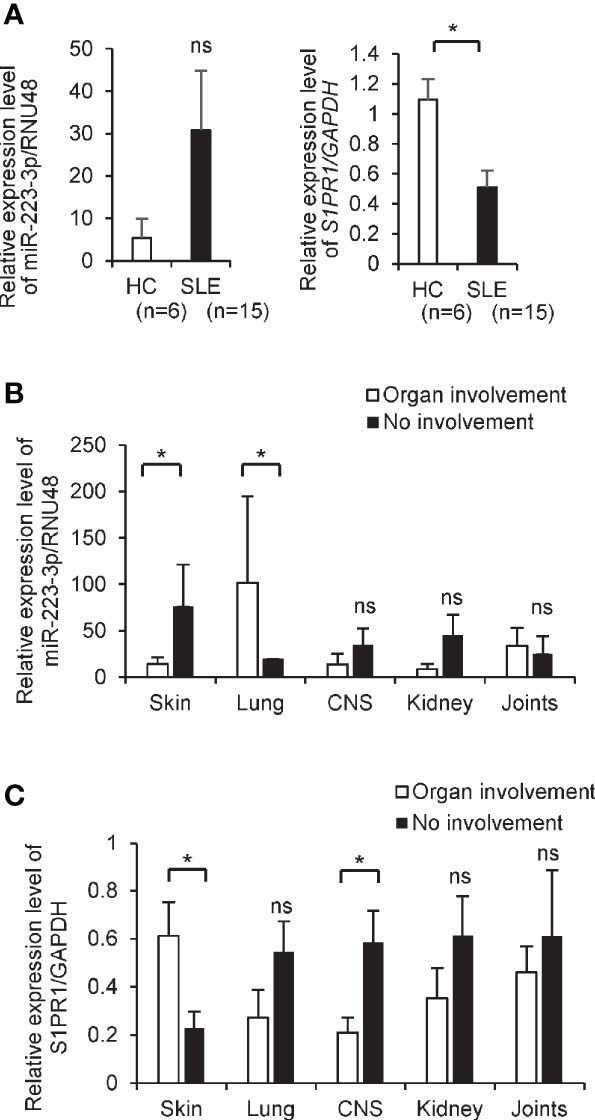
The decreased level of *S1PR1* and increased level of miR-223-3p transcripts in CD4^+^ T cells from SLE patients. **(A)** mRNA expression of *S1PR1* and miR-223-3p in CD4^+^ T cells isolated from healthy (n = 6) and the patients with SLE (n = 15). Data are presented as mean ± SEM. *p < 0.05, ns, not significant, by Wilcoxon signed-rank test. **(B, C)** The miR-223-3p and *S1PR1* transcript level in CD4^+^ T cells isolated from the SLE patients with organ involvement and no involvement. SLE patients with skin involvement (n = 11) and without involvement (n = 4), with lung involvement (n = 2) and without involvement (n = 13), with central nervous system involvement (n = 4) and without involvement (n = 11), with kidney involvement (n = 6) and without involvement (n = 9), with joints involvement (n = 10) and no involvement (n = 5). Data are presented as mean ± SEM. *p < 0.05, ns, not significant, by Student’s t-test.

We investigated the expression levels of *S1PR1* mRNA and miR-223-3p in circulating CD4^+^ T cells isolated from SLE patients and healthy subjects. The demographics of the enrolled SLE patients are shown in **Supplementary Table 4**. The patients with SLE also showed similar trends like MRL/lpr mice; the expression level of *S1PR1* mRNAs in CD4^+^ T cells is significantly downregulated in SLE patients than healthy control, while miR-223-3p tended to upregulate in SLE patients without statistical differences (**Figure 3A**). Given the critical role of *S1PR1* mRNA and miR-223-3p in SLE, we investigated the simple correlations with clinical parameters (**Supplementary Table 4**). miR-223-3p expression levels in SLE patients with skin disorders, such as malar rash, were significantly lower than SLE patients without skin symptoms. However, in the SLE patients with lung disorders, such as pleural effusion, miR-223-3p expression level was significantly higher than SLE patients without lung symptoms (**Figure 3B**). *S1PR1* mRNA expression level in SLE patients with skin disorder was significantly higher than SLE patients without skin involvement, while it was lower in the SLE patients with central nervous system (CNS) involvement compared with SLE patients without this manifestation (**Figure 3C**). These results indicated that miR-223-3p upregulation linked to *S1PR1* mRNA downregulation in SLE, in patients with skin involvements.

### Systemic Lupus Erythematosus Phenotypes in Mir223^−/−^Fas^lpr/lpr^ Mice

To explore the involvement of miR-223-3p in the pathogenesis of SLE, we generated B6-*Mir223*^−^*^/^*^−^*Fas^lpr/lpr^* mice. We observed B6-*Mir223*^−^*^/^*^−^*Fas^lpr/lpr^* and B6-*Mir223^+/+^Fas^lpr/lpr^* mice until 44 weeks of age and found that there were no differences in body weight, survival rate, skin score, organ weight, total cell numbers in spleen and lymph nodes, and circulating blood cell counts between two groups (**Supplementary Figures 2A–F**). The significant gain of weight was reported in B6-*Mir223*^−^*^/^*^−^*Ptprc^a/a^* compared to C57BL/6J (*Ptprc^a^*) (32) and the number of circulating neutrophils in *Mir223* knockout mice significantly increase and spontaneously develop lung inflammation marked by neutrophil infiltration (24). However, the presence of *Fas^lpr/lpr^* in mice canceled such *MiR223* deficiency-induced phenotypes. Similarly, in MRL/lpr background, no differences were observed in body weight, survival rate, skin score, organ weight, and total cell numbers in spleen and lymph nodes between MRL-*Mir223*^−^*^/^*^−^*Fas^lpr/lpr^* and MRL-*Mir223^+/+^Fas^lpr/lpr^* mice (**Supplementary Figures 3A–E**). Serum levels of total IgG and anti- dsDNA antibody were not altered in B6-*Mir223*^−^*^/^*^−^*Fas^lpr/lpr^* and B6-*Mir223^+/+^Fas^lpr/lpr^* (**Supplementary Figure 2G**). We further examined the serum levels of immunoglobulin subclasses (IgA, IgM, IgG1, IgG2b, IgG2c, and IgG3). Although IgG2b levels at 44 weeks of age significantly elevated in B6-*Mir223*^−^*^/^*^−^*Fas^lpr/lpr^*, rest of them demonstrated no significant increase (**Supplementary Figure 2G**). Similarly, there were no differences in gamma globulin and autoantibody production between MRL-*Mir223*^−^*^/^*^−^*Fas^lpr/lpr^* and MRL-*Mir223^+/+^Fas^lpr/lpr^* mice (**Supplementary Figure 3F**).

### Changes in Lymphocyte Population in Spleen and Lymph Nodes From Mir223^−/−^Fas^lpr/lpr^ Mice

**Figure 4:**
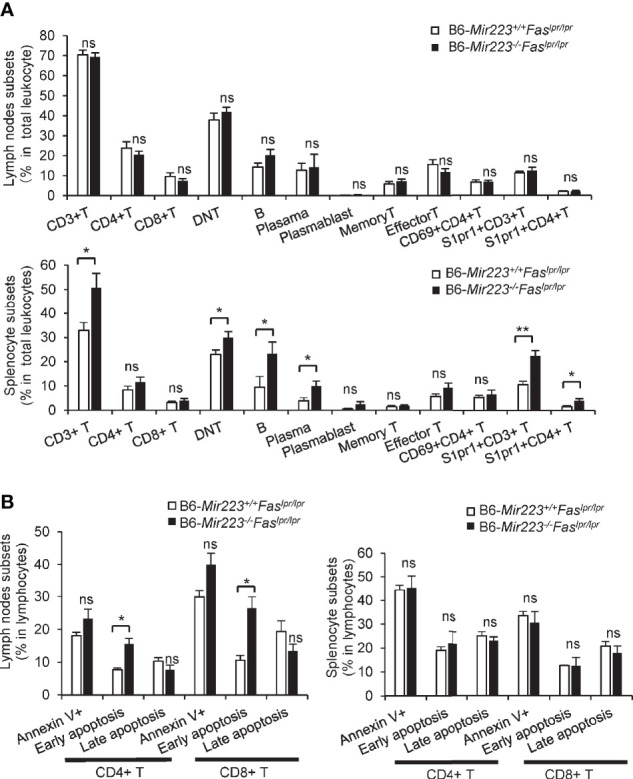
The proportion of CD3^+^ T cells, CD3^+^CD4^-^CD8^-^ T cells, CD19^+^ B cells, CD19^-^CD138^+^ cells (Plasma cells), CD3^+^S1PR1^+^ T cells and CD3^+^CD4^+^S1PR1^+^ T cells in spleen and early apoptotic cells in CD4^+^ and CD8^+^ T cells in lymph nodes were significantly increased in B6-*Mir223*^−^*^/^*^−^*Fas^lpr/lpr^* mice. **(A)** Various cellular subsets in lymph nodes and spleen. CD3^+^CD4^+^ (CD4 T cells), CD3^+^CD8^+^ (CD8 T cells), CD3^+^ CD4^−^CD8^−^ (Double negative (DN) T cells), CD19^+^ (B cells), CD19^−^CD138^+^ (Plasma cells), CD19^+^CD138^+^ (Plasmablasts), CD4^+^CD44^+^CD62L^+^ (Memory T cells), CD4^+^CD44^+^CD62L^−^ (Effector T cells), CD69^+^CD4^+^ cells, S1PR1^+^CD3^+^ cells, S1PR1^+^CD4^+^ cells. Distribution of subsets in total cells isolated from whole cervical lymph nodes and spleen were indicated in B6-*Mir223*^−^*^/^*^−^*Fas^lpr/lpr^* (n=7) and B6-*Mir223^+/+^Fas^lpr/lpr^* (n = 8) mice. Absolute number of total splenocyte and lymph nodes cell had no difference between two genotypes (**Supplementary Figure 2E**). **(B)** Apoptotic cells in lymph nodes and spleen. Annexin V^+^7-AAD^−^ (Early apoptosis cells), Annexin V^+^7-AAD^+^ (Late apoptotic cells). Distribution of subsets in CD3^+^CD4^+^ T cells or CD3^+^CD8^+^ T cells isolated from whole cervical lymph nodes and spleen were indicated in B6-*Mir223*^−^*^/^*^−^*Fas^lpr/lpr^* (n = 3) and B6-*Mir223^+/+^Fas^lpr/lpr^* (n = 3) mice. *p < 0.05, **p < 0.01, ns, not significant, by Student’s t-test in **(A)**, by Wilcoxon signed-rank test in **(B)**.

MRL/lpr mice are characterized by increased numbers of activated CD4^+^ T cells, CD3^+^CD4^−^CD8^−^ cells (double-negative T cells; DNT), B cells, and plasma cells as compared with B6 (33, 34). We next investigated the cell population in spleen and lymph nodes by flow cytometry. The proportion of CD4^+^ and CD8^+^ T cells, memory and effector T cells, and activated CD4^+^ (CD69^+^CD4^+^) T cells in both spleen and lymph nodes did not differ between B6-*Mir223*^−^*^/^*^−^*Fas^lpr/lpr^* and B6-*Mir223^+/+^Fas^lpr/lpr^* mice (**Figure 4A**). Compared with B6-*Mir223^+/+^Fas^lpr/lpr^* mice, B6-*Mir223*^−^*^/^*^−^*Fas^lpr/lpr^* showed a significantly higher proportion of CD3^+^ T cells, DNT cells, B cells, and plasma cells in the spleen (**Figure 4A**). The S1PR1^+^CD3^+^ and S1PR1^+^CD4^+^ population in the spleen increased in B6-*Mir223*^−^*^/^*^−^*Fas^lpr/lpr^* mouse (**Figure 4A**), suggesting *Mir223* deficiency contributed to the increased cell surface expression of S1PR1. The low S1P concentration in secondary lymphoid organs and relatively higher concentrations in lymphatic fluid promotes S1PR1-dependent movement of T cells from secondary lymphoid organs back into the lymphatic circulation and then into blood (35). Next, we measured the concentration of S1P in B6-*Mir223*^−^*^/^*^−^*Fas^lpr/lpr^* and B6-*Mir223^+/+^Fas^lpr/lpr^* mice; however, there were no significant differences between two genotypes (**Supplementary Figure 2G**), suggesting that surfaced S1PR1 on splenic T cells through *Mir223* deficiency may promote to egress from lymphoid organ to blood and infiltrate to inflamed tissue. In contrast, there were no differences in T and B cell populations in the spleen and lymph nodes between *Mir223* knockout lupus-prone MRL-*Mir223*^−^*^/^*^−^*Fas^lpr/lpr^* and MRL-*Mir223^+/+^Fas^lpr/lpr^* mice (**Supplementary Figure 3E** and **Supplementary Figure 5E**).

### Gene Expression and Apoptosis in CD4^+^ and CD8^+^ T Cells From Spleen and Lymph Nodes of Mir223^−/−^Fas^lpr/lpr^ Mice

The impairment of apoptosis due to *Fas* mutation is one of the mechanisms underlying the pathogenesis of *Fas^lpr/lpr^* mice. Therefore, the induction of apoptosis has been reported to ameliorate the clinical features in the mice (36). MRL/lpr mice treated with a selective agonist of S1PR1 (KRP-203) showed enhancement of lymphocytes apoptosis in lymph nodes and reduction of T cell infiltrates in kidney (23). We further examined whether apoptosis is enhanced in the lymph nodes of B6-*Mir223*^−^*^/^*^−^*Fas^lpr/lpr^* mice. Although the population of Annexin V^+^ and late apoptotic cells were unaltered, the elevated rate of early apoptotic (Annexin V^+^ and 7-AAD^−^) CD4^+^ and CD8^+^ T cells in the lymph nodes of B6-*Mir223*^−^*^/^*^−^*Fas^lpr/lpr^* (**Figure 4B**) and MRL-*Mir223*^−^*^/^*^−^*Fas^lpr/lpr^* mice were observed (**Supplementary Figure 5B**). However, anti-apoptotic Bcl-2, Bcl-xL, and proapoptotic Caspase-3 protein expression levels in lymph nodes and spleen were not altered by the deficiency of *Mir223* (**Supplementary Figure 4 and Supplementary Figure 5C**). Since the ratio of late apoptotic CD4 T cells and the cleavage of caspase-3 was not altered, the promotion of whole apoptosis process was not confirmed as the mechanisms to explain the phenotypes in B6-*Mir223*^−^*^/^*^−^*Fas^lpr/lpr^* and MRL-*Mir223*^−^*^/^*^−^*Fas^lpr/lpr^* mice.

Although miR-223-3p in CD4^+^ and CD8^+^ T cells and B cells was barely detected in the lymph nodes and spleens of B6-*Mir223*^−^*^/^*^−^*Fas^lpr/lpr^* mice (**Supplementary Figure 6B**), the *S1pr1* mRNA and protein expression in CD4^+^ T cells from spleen and lymph nodes were not altered in B6-*Mir223*^−^*^/^*^−^*Fas^lpr/lpr^* compared with that in B6-*Mir223^+/+^Fas^lpr/lpr^* mice (**Supplementary Figures 6A, C**). The increased population of S1PR1^+^CD4^+^ cells in spleen in B6-*Mir223*^−^*^/^*^−^*Fas^lpr/lpr^* suggested that S1PR1^+^CD4^+^ T cells induced by Mir223 deficiency may migrate from lymphatic tissues to non-lymphatic inflamed tissues.

### Exacerbation of Lupus Nephritis in Mir223^−/−^Fas^lpr/lpr^ Mice

**Figure 5:**
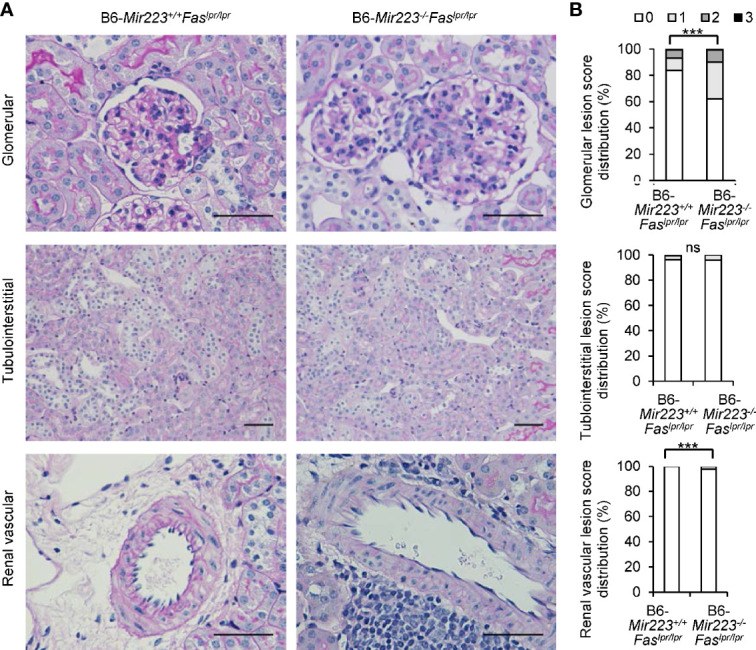
Exacerbation of lupus nephritis in B6-*Mir223*^−^*^/^*^−^*Fas^lpr/lpr^* mice. **(A)** Representative kidney sections from B6-*Mir223*^−^*^/^*^−^*Fas^lpr/lpr^* and B6-*Mir223^+/+^Fas^lpr/lpr^* mice, stained with periodic acid–Schiff (PAS). Bars = 50 µm. **(B)** Score distribution of glomerular, renal vascular and tubulointerstitial lesions in B6-*Mir223*^−^*^/^*^−^*Fas^lpr/lpr^* compared to B6-*Mir223^+/+^Fas^lpr/lpr^* mice (n = 10 per group). Data are presented as mean ± SEM. ***p < 0.001, ns, not significant, by Pearson’s χ2 test in **(B)**.

**Figure 6:**
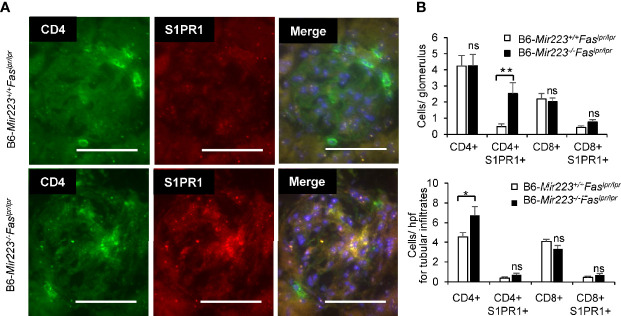
Glomerulonephritis with infiltration of CD4^+^S1PR1^+^ T cells in in B6-*Mir223*^−^*^/^*^−^*Fas^lpr/lpr^* **(A)** The image of CD4^+^ cells, CD4^+^S1PR1^+^ cells, CD8^+^ cells and CD8^+^S1PR1^+^ cells in glomerular lesion in B6-*Mir223*^−^*^/^*^−^*Fas^lpr/lpr^* compared to B6-*Mir223^+/+^Fas^lpr/lpr^* mice (n = 5 per group). Images are representative of five mice per group. Bars = 50 µm. **(B)** The number of CD4^+^ cells, CD4^+^S1PR1^+^ cells, CD8^+^ cells and CD8^+^S1PR1^+^ cells in glomerulus or tubular lesion in B6-*Mir223*^−^*^/^*^−^*Fas^lpr/lpr^* compared to B6-*Mir223^+/+^Fas^lpr/lpr^* mice (n = 5 per group). Data are presented as mean ± SEM. *p < 0.05, **p < 0.01, ns, not significant, by Student’s t-test in **(B)**.

Immune complex glomerulonephritis is the hallmark of the non-lymphatic tissue inflammation in MRL/lpr mice and human SLE patients (30, 37). In light microscopic examination of the tissue sections, an exacerbation of histologic damage in B6-*Mir223*^−^*^/^*^−^*Fas^lpr/lpr^* mice was evidenced by an expansion in glomerular size, increased cellularity, and kidney weight (**Figure 5A**, **Supplementary Figures 7C and 8B**), although mesangial matrix area demonstrated no significant difference between two genotypes (**Supplementary Figure 7C**). The distribution of glomerular and renal vascular lesions with grading scores in B6-*Mir223*^−^*^/^*^−^*Fas^lpr/lpr^* mice significantly exacerbated (**Figure 5B**). Immunofluorescence study showed S1PR1^+^CD4^+^ T cells in glomerular lesion and CD4^+^ T cells in interstitial fibrosis lesion were significantly increased in B6-*Mir223*^−^*^/^*^−^*Fas^lpr/lpr^* compared to B6-*Mir223^+/+^Fas^lpr/lpr^* mice (**Figures 6A, B**, **Supplementary Figure 8C**). We also assessed the effect of *Mir223* deficiency on glomerular immune complex formation/deposition. We found an enhanced deposition of C3 in the glomerular immune complex in B6-*Mir223*^−^*^/^*^−^*Fas^lpr/lpr^* mice compared to B6-*Mir223^+/+^Fas^lpr/lpr^* littermates (**Supplementary Figures 7A, B**) and the deposition of IgG demonstrated no significant changes (**Supplementary Figure 7B**). The tendency for the increase of S1PR1^+^CD4^+^ T cells in glomerular lesions and CD4^+^ T cells in interstitial fibrosis lesions was seen in MRL-*Mir223*^−^*^/^*^−^*Fas^lpr/lpr^* mice (**Supplementary Figures 9** and **10**). The daily urinary protein excretions tended to be higher in B6-*Mir223*^−^*^/^*^−^*Fas^lpr/lpr^* than B6-*Mir223^+/+^Fas^lpr/lpr^* mice (**Supplementary Figure 8A**).

Chemokines contribute to renal damage by recruiting inflammatory cells to the kidney. The CXCR3 ligands, CXCL9 and CXCL10 were expressed at high levels at early time points in the spleen and CCL-2, CCL4, CCL5, CXCL10 in nephritic kidneys of *Fas^lpr/lpr^* mice (38–41). T cell migration from blood into tissue is induced by chemokines CXCL9 and CXCL11 presented on the endothelial surface, which activates surface expression of S1PR1 and S1PR4 on T cells (42). The increased S1P present in inflamed peripheral tissues may further induce T cell retention. Therefore, we next evaluated the chemokine mRNA levels in renal cortex. In both B6 and MRL/lpr mice, *Cxcl10, Cxcl11 and Ccl2* demonstrated higher mRNA levels in *Mir223*^−^*^/^*^−^*Fas^lpr/lpr^* compared with *Mir223^+/+^Fas^lpr/lpr^*; however, it did not reach statistical differences (**Supplementary Figures 8D** and **11**). Although *Cxcl10* was one of the predicted target mRNAs of miR-223-3p from miRDB (http://mirdb.org.), the roles of chemokines in infiltration of S1PR1+CD4+ T cells in *Mir223*^−^*^/^*^−^*Fas^lpr/lp^* may be limited.

Taken together, *Mir223* deficiency can exacerbate the functional and pathologic damage to the kidneys in B6-MRL-Fas^lpr^ mice by facilitating the infiltration of S1PR1^+^CD4^+^ T cells in both glomerular and interstitial lesions.

## Discussion

Recent studies have demonstrated that miRNAs play important roles in the pathogenesis of SLE (3). In pristine-induced lupus mice, lupus nephritis is ameliorated by miR-654 mimic injection therapy (43). *Mir155* knockout mice demonstrated the amelioration of autoimmune inflammation (5). miRNAs themselves might become therapeutic modalities in the SLE treatment, as seen in the cancer therapies (44). In the current investigation, we focused on *S1pr1* as a new target of miR-223-3p. In transgenic mice with the persistent expression of human S1PR1 in lymphocytes, the activated T cells demonstrated decreased entry into lymph nodes and increased entry into circulation (45). However, S1PR1 agonists have been shown to prevent T cell migration from lymph nodes into circulation and result in amelioration of the clinical features of SLE in model mice (18, 19, 21–23). After phosphorylation by sphingosine kinases, fingolimod, S1PR1 modulator, functionally antagonizes S1PR1 expressed on lymphocytes by receptor internalization and degradation. This process prevents a functionally normal response to the endogenous S1P gradient, thereby blocking lymphocytes to egress from secondary lymphoid organs to the blood and reducing the circulation of autoreactive lymphocytes (46). Therefore, S1PR1 dysfunction and overexpression in T cells are hypothesized to be linked to the pathogenesis of SLE.

In B6-*Mir223*^−^*^/^*^−^*Fas^lpr/lpr^* mice, the population of splenic S1PR1^+^CD4^+^ T cells was significantly increased compared with B6-*Mir223^+/+^Fas^lpr/lpr^* mice and thus S1PR1^+^CD4^+^ T cells were induced by *Mir223* deficiency. Although *S1pr1* mRNA and protein expression was not altered in isolated splenic CD4^+^ T cells from B6-*Mir223*^−^*^/^*^−^*Fas^lpr/lpr^* mice compared with B6-*Mir223^+/+^Fas^lpr/lpr^* mice, S1PR1^+^CD4^+^ T cells were actively infiltrated into the glomeruli in B6-*Mir223*^−^*^/^*^−^*Fas^lpr/lpr^* mice. The results suggested that S1PR1^+^CD4^+^ T cells induced by *Mir223* deficiency preferentially migrated into spleen and inflamed kidney tissues. We postulate that *Mir223* is a new therapeutic target in the treatment of SLE by modulating the expression of S1PR1.

Since *Mir223* is highly expressed in granulocytes compared to T and B cells (24), we should consider the functional roles of *Mir223* in various immune-mediated cells in the translational research. *Mir223* is essential for innate immune responses and inflammatory diseases such as rheumatoid arthritis and inflammatory bowel disease (IBD) (47, 48). *Mir223* is overexpressed in fibroblast-like synoviocytes, and synovial fluid of the patients with rheumatoid arthritis and lentivirus-mediated silencing of *Mir223* can suppress collagen-induced arthritis in mice by decreasing macrophage colony-stimulating factor receptor levels in the synovium (49). Of interest, in *Mir223* deficient mice, granulocyte numbers are increased, albeit with an abnormal phenotype, and spontaneously develop inflammatory lung pathology due to hyperactivity of the neutrophils (24). Indeed, neutrophil numbers in peripheral blood tended to be higher in B6-*Mir223*^−^*^/^*^−^*Fas^lpr/lpr^* mice compared to B6-*Mir223^+/+^Fas^lpr/lpr^* mice (**Supplementary Figure 2E**). Neutrophils in SLE have abnormal functions like reducing phagocytosis capabilities and apoptotic pathways and increasing oxidative activity and NETosis (50). These neutrophils infiltrate into kidney tissues affected by lupus nephritis (51). Although *Mir223* is not expressed in kidney cells and there were no renal damages in *Mir223* deficient mice (24), *Mir223* deficient neutrophils were hyperactive and assumed to contribute the glomerular injuries in *Mir223*^−^*^/^*^−^*Fas^lpr/lpr^* mice. Overlapping abnormality in neutrophils by *Mir223* deficiency and SLE was thought to be one of the causes of exacerbation of lupus nephritis in our B6-*Mir223*^−^*^/^*^−^*Fas^lpr/lpr^* mice.

In addition to the roles in the granulocytes, *Mir223* has been known to be functional particularly in CD4^+^Th17 cells, which exacerbated the experimental autoimmune uveitis (EAU) by promoting autoreactive Th17 cell responses by inhibiting transcription factor FOXO3 expression (52). CD4^+^Th17 cells were also shown to be increased in SLE patients and MRL/lpr mice (53, 54) and they infiltrate into the kidney tissues and contribute to tissue damage by producing IL-17 and IFN-*γ* (55). CD4^+^ T cells also infiltrate in kidney tissues and link to exacerbation of lupus nephritis (23). We initially hypothesized that *Mir223* deficiency may ameliorate lupus nephritis since *MIR223* is overexpressed in CD4^+^ T cells in the patients with relapsing multiple sclerosis and rheumatoid arthritis (9, 56). However, *Mir223* deficiency exacerbated glomerulonephritis in B6-*Mir223*^−−^*^-^Fas^lpr/lpr^* mice by facilitating the infiltration of S1PR1^+^CD4^+^ T cells into kidney tissues.

Although the glomerular deposition of C3 was increased in B6-*Mir223*^−^*^/^*^−^*Fas^lpr/lpr^* mice, there were no differences in the deposition of IgG (**Supplementary Figure 7B**). The complement system is composed of three major arms of activation pathways and plays protective and pathogenic roles in the development of SLE. The classical pathway (CP) contributes to the clearance of immune complexes and apoptotic cells, whereas the alternative pathway (AP) and lectin pathway (LP) in lupus exacerbates renal inflammation (57). Activation of LP is initiated by pattern recognition molecules (PRMs) without antibodies, and they are composed of mannose-binding lectin (MBL), ficolin, collectin-liver 10 (CL-10), and CL-11 (58). MBL-associated serine proteases-1 and -2 (MASP-1 and MASP-2) are the enzymatic constituents of the LP and form a complex with the PRMs. *Masp1* knockout lupus-prone MRL/*lpr* mice (*Masp1/3−/−* MRL/*lpr* mice) lacking both MASP-1 and its splicing isoform MASP-3 demonstrated reduced activation of LP and AP. In this model, there were no significant differences in glomerular IgG but significantly reduced glomerular C3 deposition compared to their wild-type littermates (57). In B6-*Mir223*^−^*^/^*^−^*Fas^lpr/lpr^* mice, we can speculate that the infiltration of S1PR1^+^CD4^+^ T cells may induce the cellular damage of the glomeruli, the release of damage-associated molecular patterns (DAMPs) and subsequent activation of LP (59).

There are some limitations in the current investigation. First, we employed total *Mir223* knockout mice for the investigation. The CD4^+^ T cell specific *Mir223* knockout mice is required to further confirm the findings observed in this study. In addition, we compared miRNA and mRNA profiling between B6 and MRL/lpr in the initial screening of the candidate miRNAs (**Figure 1**). MRL/MpJ mice are the parent and control strain for MRL/lpr, but we did not compare the expression of *Mir223* and *S1pr1* between these strains. The genetic background B6/lpr and MRL/lpr also influenced the phenotype of the Mir223 knockout mice. However, all other functional gene knockout studies were performed under identical genetic backgrounds such as B6 or MRL/lpr. Second, we did not employ S1PR1 antagonist to confirm the relationship between *S1pr1* and *Mir223*. S1PR1-5 antagonist (FTY720/fingolimod) is approved for multiple sclerosis (60), while selective S1PR1 antagonist (cenerimod) is currently under phase 2 clinical study (NCT-02472795) in the patients with SLE. Cenerimod has been reported to ameliorate systemic and organ-specific pathology and inflammation in MRL/lpr mice (61), and the administration of cenerimod into *Mir223*^−^*^/^*^−^*Fas^lpr/lpr^* mice should be investigated in future studies.

In conclusion, we presented that the deletion of *Mir223* exacerbated the lupus phenotypes associated with increased population of S1PR1^+^CD4^+^ T cells and their enhanced infiltration in inflamed kidney tissues. In addition to the modulation of the function of S1PR1, *Mir223* may be a valid therapeutic modality in the treatment of SLE by targeting S1PR1^+^CD4^+^ T cells. We believe that the current investigation provides novel data pertaining to the T cell circulation in SLE.

## Data Availability Statement

All raw and processed data are freely accessible in the Gene Expression Omnibus (https://www.ncbi.nlm.nih.gov/geo/) under the accession number GSE87219.

## Ethics Statement

The studies involving human participants were reviewed and approved by the Ethical Committee, Okayama University Hospital. The patients/participants provided their written informed consent to participate in this study. The animal study was reviewed and approved by the Animal Care and Use Committee of the Department of Animal Resources, Advanced Science Research Center, Okayama University.

## Author Contributions

HA, SW, and JW designed and conceptualized the study. HA, SW, SZ, EK, TM, YM, and JW performed the experiments and were involved in the data acquisition. HA and JW analyzed and interpreted the data. All authors contributed to the article and approved the submitted version.

## Funding

This work was supported by JSPS Grant-in-Aid for Scientific Research, Grant numbers (16K09895, 16K09896, 16K19601, 16K19602, 16K19600, 17K09976, 18K16151, and 20K17442), the KAWASAKI Foundation for Medical Science and Medical Welfare, Research Project Grant from Kawasaki Medical School (R02S005), and GSK Japan Research Grant 2020.

## Conflict of Interest

SH-A and TM receive scholarship donations from Chugai and Ayumi. KS-W receives speaker honoraria from Chugai. YM receives scholarship donations from Chugai and Ayumi and speaker honoraria from Eli Lilly. JW receives speaker honoraria from Astra Zeneca, Daiichi Sankyo, MSD, Novartis, Tanabe Mitsubishi, Taisho Toyama and receives grant support from Baxter, Chugai, Dainippon Sumitomo, Ono, and Teijin.

The remaining authors declare that the research was conducted in the absence of any commercial or financial relationships that could be construed as a potential conflict of interest.
