# Supplementary material for: Deletion of Mir223 Exacerbates Lupus Nephritis by Targeting S1pr1 in Faslpr/lpr Mice
Source: Front Immunol. 2021 Jan 26;11:616141. doi: 10.3389/fimmu.2020.616141 (PMC7871001; doi:10.3389/fimmu.2020.616141)

Supplementary Materials and Methods

Generation of B6-Mir223$^{-/-}$Fas$^{lpr/lpr}$ and MRL-Mir223$^{-/-}$Fas$^{lpr/lpr}$ mice

Using standard breeding techniques, we generated B6-*Mir223$^{-/-}$Fas$^{lpr/lpr}$* mice by crossing B6-*Fas$^{lpr/lpr}$Ptprc$^{b/b}$* males with B6-*Mir223$^{-/-}$Ptprc$^{a/a}$* females, then backcrossing B6-*Mir223$^{+/-}$Fas$^{lpr/wt}$Ptprc$^{a/b}$* F1 females to B6-*Fas$^{lpr/lpr}$Ptpr$^{b/b}$* males. *Mir223$^{-/y}$Fas$^{lpr/lpr}$Ptprc$^{b/b}$* F2 males and *Mir223$^{+/-}$Fas$^{lpr/lpr}$Ptprc$^{b/b}$* F2 females were intercrossed to obtain the *Mir223$^{-/-}$Fas$^{lpr/lpr}$Ptprc$^{b/b}$* (B6-*Mir223$^{-/-}$Fas$^{lpr/lpr}$*) females (n=15). *Mir223$^{+/y}$Fas$^{lpr/lpr}$Ptprc$^{b/b}$* F2 males and *Mir223$^{+/-}$Fas$^{lpr/lpr}$Ptprc$^{b/b}$* F2 females were also intercrossed to obtain B6-*Mir223$^{+/+}$Fas$^{lpr/lpr}$Ptprc$^{b/b}$* (B6-*Mir223$^{+/+}$Fas$^{lpr/lpr}$*) female mice (n=15). Mice genotypes of lpr, Ptprc and Mir223 mutation were identified by PCR with DNA obtained by tail biopsy. These mice were further monitored until the age of 44 weeks. Sera were collected at 12 and 44 weeks of age, while weight and proteinuria were assessed from 12 weeks of age and every 4 weeks thereafter. Tissue samples, including spleen, lymph nodes, and kidneys, were obtained at the end of the study.

Similarly, we generated MRL-*Mir223$^{-/-}$Fas$^{lpr/lpr}$* mice by crossing MRL-*Fas$^{lpr/lpr}$Ptprc$^{b/b}$* males with B6-*Mir223$^{-/-}$Ptprc$^{a/a}$* females to yield MRL-*Mir223$^{+/-}$Fas$^{lpr/wt}$Ptprc$^{a/b}$* F1 females. They were further backcrossed with MRL-*Fas$^{lpr/lpr}$Ptprc$^{b/b}$* males for 8 generations. Finally, MRL-*Mir223$^{-/y}$Fas$^{lpr/lpr}$Ptprc$^{b/b}$* F9 males and MRL-*Mir223$^{+/-}$Fas$^{lpr/lpr}$Ptprc$^{b/b}$* F9 females were intercrossed to obtain MRL-*Mir223$^{-/-}$Fas$^{lpr/lpr}$Ptprc$^{b/b}$* (MRL-*Mir223$^{-/-}$Fas$^{lpr/lpr}$*) females (n=10). *Mir223$^{+/y}$Fas$^{lpr/lpr}$Ptprc$^{b/b}$* F9 males and *Mir223$^{+/-}$Fas$^{lpr/lpr}$Ptprc$^{b/b}$* F9 females were also intercrossed to obtain the *Mir223$^{+/+}$Fas$^{lpr/lpr}$Ptprc$^{b/b}$* (MRL-*Mir223$^{+/+}$Fas$^{lpr/lpr}$*) females (n=10). These mice were further monitored until the age of 16 weeks. Sera were collected at 8 and 16 weeks of age,

while weight and proteinuria were assessed from 8 weeks of age and every 4 weeks thereafter. Tissue samples were obtained at the end of the observation.

Serum levels of total IgG (Abcam), isotype-specific IgGs (Southern Biotech), anti-double-stranded DNA (anti-dsDNA) (Shibayagi) and anti-S1P (Echelon Biosciences Inc.) were measured using commercially available ELISA kits in accordance with the manufacturers' instructions. The absorbance was determined using a microplate reader set at 450 nm. Urinary albumin excretions were measured every 4 weeks using LBIS Mouse Urinary Albumin Assay Kit (S-type) (SHIMA Laboratories) and standardized by urine creatinine concentration.

Isolation of mouse primary T cells and B cells

Mouse $CD4^+$ and $CD8^+$ T cells and B cells in spleen or lymph nodes were isolated using a CD4 isolation kit II (Miltenyi Biotec), CD8a isolation kit II (Miltenyi Biotec) and PanBcell isolation kit II (Miltenyi Biotec) by negative selection, respectively. A purity rate of >96.6% for isolated $CD4^+$ and $CD8^+$ T cells and B cells was confirmed by flow cytometry.

EL4 cell line culture and transfection of miRNAs

The EL4 mouse T cell line was obtained from the American Type Culture Collection and cultured in DMEM (Life Technologies) supplemented with 10% FBS (Life Technologies), 100 U/ml penicillin, and 100 μg/ml streptomycin at 37°C in a 5% CO_2 incubator. The transfection of miRNA mimics (mirVana; Invitrogen) was performed with Lipofectamine RNAiMAX (Invitrogen) by the reverse transfection procedure. EL4 cells were transfected with miR-223-3p mimic or its negative control at a final concentration of 10 nM. They

were harvested after 24 hours for RNA extraction or after 48 hours for Western blot analysis. To detect apoptotic cells, EL4 cells were stained with FITC-conjugated Annexin V and 7-aminoactinomycin D staining (7-AAD) using the FITC Annexin V Apoptosis Detection Kit with 7-AAD (640922, BioLegend) following the manufacturer's recommendation. The stained cells were directly analyzed with FACSAria I (BD Biosciences) and FlowJo software (TreeStar).

Western blot analysis

The lysates of murine splenic $CD4^+$ T cells and EL4 cells which were transfected with miR-223-3p mimic or its negative control were prepared by homogenization with CelLytic M (Sigma-Aldrich), supplemented with a protease inhibitor cocktail (Sigma-Aldrich). Protein concentration was determined using the Pierce BCA protein Assay kit (Thermo Fisher Scientific). They were run on a Criterion™TGX™Precast Gel (Bio-Rad) electrophoresis (SDS-PAGE) and blotted on Amersham Hybond P 0.45 μm PVDF (GE Healthcare Life Sciences). The primary antibodies against S1PR1 at 1:500 dilution (ab137467, Abcam), GAPDH at 1:1,000 (5174T, Cell Signaling), Bcl-xL at 1:1000 (2762S, Cell Signaling), Bcl2 at 1:1000 (3498S, Cell Signaling), Caspase-3 at 1:1000 (14220S, Cell signaling) and β-actin at 1:4000 (A2066; Sigma-Aldrich) were incubated overnight at 4°C in TBS supplemented with 0.1% Tween 20 (TBS-T) containing 5% bovine serum albumin. Then, the blots were washed with TBS-T and incubated with Goat anti-rabbit IgG-HRP at 1:20,000 dilution (7074S, Cell Signaling) for 60 minutes at room temperature. After three-time washes with TBS-T, they were developed using the SuperSignal™ West Femto Maximum Sensitivity Substrate (Thermo Fisher Scientific)

and visualized using an ImageQuant LAS 4000 Mini kit (GE Healthcare Life Sciences). Protein bands were semiquantified by densitometry using Image J.

Isolation of ubiquitinated proteins and Western blot

Isolation of ubiquitin-conjugated proteins was performed using the UbiQapture-Q Kit (Enzo Life Science), as described by the manufacturer. A total of 25 µg of lysates from cells was used per assay. Samples were added to the tubes containing 20 µl UbiQapture-Q matrix and incubated overnight at 4°C in a horizontal rotor mixer. The matrix was then carefully washed and unbound fraction samples were kept for analysis. Finally, the ubiquitin-protein conjugates were eluted by addition of 100 µl sample buffer and heating at 95°C for 10 min. The elution fraction was clarified from the matrix. The starting material, unbound and elution fraction samples were analyzed by immunoblotting with the anti S1PR1 antibody and anti-monoubiquitinated and anti-polyubiquitinated conjugate antibodies (FK2) (Enzo Life Science).

Flow cytometry analysis

The spleen and cervical lymph nodes from MRL/lpr mice were finely minced and incubated in a fresh digestion medium containing 3 ml DMEM with 2 mg/ml collagenase D (Roche) at 37°C for 30 minutes, with gentle shaking at 200 rpm on an orbital shaker. Single-cell suspensions were prepared from the spleens and lymph nodes of MRL-*Mir223*$^{-/-}$*Fas*$^{lpr/lpr}$, MRL-*Mir223*$^{+/+}$*Fas*$^{lpr/lpr}$, B6-*Mir223*$^{-/-}$*Fas*$^{lpr/lpr}$ and B6-*Mir223*$^{+/+}$*Fas*$^{lpr/lpr}$ mice. Red blood cells were removed using a red cell lysing buffer (BD Pharm Lyse). The cells were first incubated at 4°C for 10 minutes with an Fc receptor (FcR) blocking reagent (Miltenyi Biotec) to reduce nonspecific binding of antibodies to

FcRs. The 1×10^6 cells derived from the spleen and lymph nodes were incubated at 4°C for 30 minutes in staining buffer (BioLegend) with the relevant optimized amount of fluorochrome conjugated antibodies or the appropriate isotype controls: FITC-conjugated anti CD4 (GK1.5), VioBlue-conjugated anti-CD4 (GK1.5), allophyocyanin (APC)-conjugated anti-CD8 (53-6.7), APC-conjugated anti-CD62L (MEL14-H2.100), phycoerythrin (PE)-conjugated anti-CD44 (IM7.8.1), APC-conjugated anti-CD69 (H1.2F3), APC-conjugated anti-CD19 (6D5), PE-conjugated anti-CD138 (clone REA104). All antibodies were purchased from Miltenyi Biotec except BV421-conjugated anti-CD3e (SK7) (BD Biosciences) and APC-conjugated anti-S1pr1 (713412) (R&D Systems). Dead cells were excluded from the analysis using 7-AAD staining (BD PharMingen). All data were acquired with a FACSAria I flow cytometer (BD Biosciences) and analyzed using FlowJo software (TreeStar).

Supplementary Table1. miRNAs upregulated in CD4$^+$ T cells derived from MRL/lpr mice compared with B6 mice.

1. RPM：Reads per million mapped reads.

2. Fold change＝ MRL/lpr RPM/B6 RPM

The candidate miRNAs are sorted by fold change.

miRNAs	Total Reads	B6 Reads	MRL/lpr Reads	B6 RPM[1]	MRL/lpr RPM[1]	Fold change[2]
mmu-miR-144-3p	1143	13	1130	0.39	37.70	97.5
mmu-miR-451a	383	11	372	0.33	12.41	37.9
mmu-miR-582-3p	104	4	100	0.12	3.34	28.0
mmu-miR-223-3p	6706	267	6439	7.94	214.84	27.0
mmu-miR-143-3p	4138	179	3959	5.32	132.09	24.8
mmu-miR-127-3p	16908	817	16091	24.29	536.89	22.1
mmu-miR-144-5p	1772	88	1684	2.62	56.19	21.4
mmu-miR-152-3p	286	15	271	0.45	9.04	20.2
mmu-miR-434-5p	136	8	128	0.24	4.27	17.9
mmu-miR-411-5p	2527	165	2362	4.91	78.81	16.0
mmu-miR-410-3p	1175	77	1098	2.29	36.64	16.0
mmu-miR-21a-3p	2481	165	2316	4.91	77.27	15.7
mmu-miR-300-3p	207	14	193	0.42	6.44	15.4
mmu-miR-381-3p	915	73	842	2.17	28.09	12.9
mmu-miR-541-5p	1157	97	1060	2.88	35.37	12.2
mmu-miR-3107-5p	151687	12909	138778	383.80	4630.41	12.0
mmu-miR-486-5p	151687	12909	138778	383.80	4630.41	12.0
mmu-miR-183-5p	6390	571	5819	16.98	194.15	11.4
mmu-miR-379-5p	121	11	110	0.33	3.67	11.2

Supplementary Table 2. mRNA profiling in splenic CD4+ T cells purified in MRL/lpr lupus-prone mice and B6 mice. Predicted target genes of differentially expressed miR-223-3p in miRDB.

1. FPKM：Reads per kilobase of exon model per million mapped reads.
2. Fold change＝$FPKM_{MRL/lpr}/FPKM_{B6}$

Gene	FPKM B6	FPKM MRL	Fold change	p_value
Trim30d	5.45455	0.225717	0.041381507	0.00037222
Dusp10	184.754	22.679	0.122752133	0.000072097
Atp1b1	25.9197	5.39045	0.207967501	0.0260443
Kctd4	0.0513919	0.0109806	0.213663148	0.904441
Scn3a	0.0154045	0.00329138	0.213663148	0.263443
S1pr1	138.017	33.0183	0.239233279	0.00477601
Ttc28	6.13654	1.57781	0.257117551	0.0280216
Smoc1	0.310147	0.0852	0.274707842	0.211336
Apold1	0.0656456	0.0200373	0.305234512	0.335325
Foxp1	42.1957	14.1079	0.334342811	0.0922709
Cd8a	1.18792	0.414419	0.348859977	0.40143
Slc4a4	0.0360867	0.0128504	0.356098929	0.424302
Kmt2c	22.5525	8.07121	0.3578855	0.0400435
Mfhas1	10.5987	3.99277	0.376724039	0.0978997
Tgfbr3	11.4107	4.68328	0.410430543	0.121272
Rbm20	0.103833	0.0434059	0.418036263	0.398298
Acpp	7.70997	3.23427	0.419493395	0.306921
Slc25a53	3.38412	1.47201	0.434976691	0.532931
Tbc1d16	0.901121	0.408687	0.453530035	0.379875
Il6st	30.1119	14.1055	0.468437994	0.1333
Siah1a	12.0616	5.90726	0.489757673	0.328482
Ranbp3l	0.105077	0.0518103	0.493068502	0.513081
Adcy1	0.0358616	0.0178788	0.498549963	0.513053

Supplementary Table 3. Human and mouse miR-223 binding site of *S1PR1*

Mus *S1pr1* 3'-UTR	5′-ACUCCCCAA AAGCAUUACU UUAACUGACA GGGAACAUCA-3′
Mus miR-223	3′-AC CCCAUAAACU GUUUGACUGU-5′
Homo *S1PR1* 3'-UTR	5′-UAUCAACUUU UAAACAUUAA UAAACUGAUU UUUUUAAAGA-3′
Homo miR-223	3′-AC CCCAUAAACU GUUUGACUGU-5′

Red shows the seed sequence of miR-223.

Supplementary Table 4. Demographics of the patients with systemic erythematosus (SLE).

Parameters	SLE (n=15)<br>Mean±standard deviation
Sex (male / female)	5/10
Race	Japanese
Age (years)	46±15.1
SLEDAI (systemic erythematosus disease activity index)	17.1±8.5
BILAG (British Isles Lupus Assessment Group disease activity index)	14.5±4.9
Anti-nuclear antibody (ANA) positive (%)	93.3
ANA titer (x)	828.6±638.3
dsDNA antibody positive (%)	86.7
Anti-dsDNA antibody (IU/mL)	134.4±144.0
Anti-Sm positive (%)	26.7
Anti-Sm antibody (U/ml)	20.4±42.4
Anti-ribonucleoprotein (RNP) positive (%)	40.0
Anti-RNP antibody (U/mL)	81.6±108.9
Anti-cardiolipin (CL) antibody (%)	33.3
Anti-CL antibody (U/mL)	18.4±28.4
Aniti-β2 glycoprotein I (β2GPI) positive (%)	6.7
Aniti-β2GPI antibody (U/mL)	3.8±9.9
Lupus anticoagulant positive (%)	40.0
Rheumatoid factor (RF) (IU/mL)	11.2±11.6
Anti-SS-A positive (%)	80.0
Anti-SS-A antibody (U/mL)	159.7±105.2
Anti-SS-B positive rate (%)	13.3
Anti-SS-B antibody (U/mL)	16.0±44.2
IgG (mg/dL)	2255.6±504.3
IgA (mg/dL)	338.1±145.9
IgM (mg/dL)	161.7±131.0
C3 below normal range (%)	80.0
C3 titer (mg/dL)	44.6±20.6
C4 below normal range (%)	93.3
C4 titer (mg/dL)	7.0±4.2
CH50 below normal range (%)	93.3
CH50 (U/mL)	17.2±10.7
Serum creatinine (mg/dL)	0.7±0.2
Urine protein / creatinine (g/gCr)	2.1±3.9
White blood cells ($\times 10^3/\mu L$)	3304.0±1531.6
Lymphocytes ($\times 10^3/\mu L$)	861.3±1037.0
Hemoglobin (g/dL)	11.3±1.8
Platelets ($\times 10^4/\mu L$)	16.0±7.1
Lupus nephritis (%)	40.0
Arthritis (%)	66.7
Skin rash (%)	73.3
Neuropsychiatric SLE (%)	20.0

Supplementary Table 5. The list of the primers used for RT-PCR. Genes analyzed by Taqman Gene Expression Assays and Taqman MicroRNA Assays.

Gene Name	Probe Number
S1pr1	Mm02619656_s1
Cxcl9	Mm00434946_m1
Cxcl10	Mm00445235_m1
Cxcl11	Mm00444662_m1
Ccl2	Mm00441242_m1
Ccl4	Mm00443111_m1
Ccl5	Mm01302427_m1
Gapdh	Mm99999915_g1
S1PR1	Hs01922614_s1
GAPDH	Hs02786624_g1
miR-223-3p	002295
snoRNA202	001232
snoRNA234	001234
RNU48	001006

Supplementary Figure 1. The protein expression of *S1pr1* gene in $CD4^+$ T cells isolated from C57BL/6 (B6) and MRL/lpr lupus-prone (MRL/lpr) mice. (**A)** Western blot analyses of S1PR1 in $CD4^+$ T cells in B6 and MRL/lpr mice. Band intensity (normalized to total β -actin) measured by using ImageJ software. (**B)** $CD4^+$ T cells in B6 and MRL/lpr mice were lysed in CelLytic M and subjected to UbiQapture-Q matrix. The matrix beads were washed, and supernatants were kept as unbound fraction (UF). The polyubiquitinated proteins were eluted. Starting material (SM), UF, and elution fraction (EF) were blotted with rabbit mouse anti-monoubiquitinated and anti-polyubiquitinated conjugate antibodies (FK2) and anti S1PR1 antibody**.**

Supplementary Figure 2. *Mir223* knockout lupus-prone B6.MRL-Fas^{lpr} mice (B6-$Mir223^{-/-}Fas^{lpr/lpr}$) showed no significant change in survival rate, organ weights and skin scores. (A) Body weight (B) survival rates (**C)** skin score (**D).** organ weights at 44 weeks in B6-$Mir223^{-/-}Fas^{lpr/lpr}$ (n=16) and B6-$Mir223^{+/+}Fas^{lpr/lpr}$ (n=17) mice. (**E)** Splenocytes cell counts and lymph nodes cell counts at 44 weeks in B6-$Mir223^{-/-}Fas^{lpr/lpr}$ (n=7) and B6-$Mir223^{+/+}Fas^{lpr/lpr}$ (n=8) mice. **(F)** Peripheral blood counts of B6-$Mir223^{-/-}Fas^{lpr/lpr}$ (n=10) and B6-$Mir223^{+/+}Fas^{lpr/lpr}$ (n=9) mice. **(G)** Serum levels of total IgG, anti-double-stranded DNA antibodies (anti-dsDNA Ab), S1P concentration and each immunoglobulin subclass at 12 and 44 weeks in B6-$Mir223^{-/-}Fas^{lpr/lpr}$ (n=7) and B6-$Mir223^{+/+}Fas^{lpr/lpr}$ (n=8) mice were measured by enzyme-linked immunosorbent assay. Data are presented as mean ± SEM. $*p < 0.05$, $***p < 0.001$, ns = not significant, by Student's t-test in (**C), (D), (E), (F)** and (**G)**, by Wilcoxon signed-rank test in (**A)**, and by log-rank test in (**B)**.

Supplementary Figure 3. *Mir223* knockout lupus-prone MRL/MpJ-Fas^{lpr} /J mice (MRL-$Mir223^{-/-}$$Fas^{lpr/lpr}$) showed no significant change in survival rate, organ weights and skin scores. (**A)** Body weight (**B)** survival rates (**C)** skin score (**D)** organ weights at 16 weeks in MRL-$Mir223^{-/-}$$Fas^{lpr/lpr}$ (n=11) and MRL-$Mir223^{+/+}$$Fas^{lpr/lpr}$ (n=11) mice. (**E)** Splenocytes cell counts and lymph nodes cell counts at 16 weeks in MRL-$Mir223^{-/-}$$Fas^{lpr/lpr}$ (n=4) and MRL-$Mir223^{+/+}$$Fas^{lpr/lpr}$ (n=4) mice. (**F)** Serum levels of total IgG, anti-double-stranded DNA antibodies (anti-dsDNA Ab), S1P concentration and each immunoglobulin subclass at 8 and 16 weeks in MRL-$Mir223^{-/-}$$Fas^{lpr/lpr}$ (n=10) and MRL-$Mir223^{+/+}$$Fas^{lpr/lpr}$ (n=7) mice were measured by enzyme-linked immunosorbent assay. Data are presented as mean ± SEM. $**p < 0.01$, ns = not significant, by Student's t-test in (**C), (D), (E) and (F**), by Wilcoxon signed-rank test in **(A)** , and by log-rank test in (**B**).

Supplementary Figure 4. The apoptosis related proteins expression in B6-$Mir223^{-/-}$$Fas^{lpr/lpr}$mice. Western blot analyses of Bcl-xL, Bcl-2 and Caspase-3 in axillary lymph nodes and spleen B6-$Mir223^{-/-}$$Fas^{lpr/lpr}$ (n=3) and B6-$Mir223^{+/+}$$Fas^{lpr/lpr}$ (n=3) mice. Band intensity (normalized to total β -actin) measured by using ImageJ software. Quantification of Western blot results (below). (n=3 per group). Data are presented as mean ± SEM. ns = not significant, by Wilcoxon signed-rank test.

Supplementary Figure 5. Lymphocyte subsets in splenocytes and cervical lymph nodes cells in MRL-$Mir223^{-/-}$$Fas^{lpr/lpr}$ and MRL-$Mir223^{+/+}$$Fas^{lpr/lpr}$ mice. (**A)** Various cellular subsets in lymph nodes and spleen. $CD3^+CD4^+$ (CD4 T cells), $CD3^+CD8^+$ (CD8 T cells),

$CD3^+CD4^-CD8^-$ (Double negative (DN) T cells), $CD19^+$ (B cells), $CD19^-CD138^+$ (Plasma cells), $CD4^+CD44^+CD62L^+$ (Memory T cells), $CD4^+CD44^+CD62L^-$ (Effector T cells), $CD69^+CD4^+$ cells, $S1PR1^+CD3^+$ cells, $S1PR1^+CD4^+$ cells. Distribution of cellular subsets in total cells isolated from whole cervical lymph nodes and spleen were indicated in MRL-*Mir223*$^{-/-}$*Fas*$^{lpr/lpr}$ (n=4) and MRL-*Mir223*$^{+/+}$*Fas*$^{lpr/lpr}$ (n=4) mice. Absolute number of total splenocyte and lymph nodes cell had no difference between two genotypes (**Supplementary Figure 3E**). (**B)** Apoptotic cellular subsets in lymph nodes and spleen. Annexin V^+7-AAD^- (Early apoptosis cells), Annexin V^+7-AAD^+ (Late apoptosis cells). Distribution of cellular subsets in each $CD3^+CD4^+$ T cells or $CD3^+CD8^+$ T cells isolated from whole cervical lymph nodes and spleen were indicated in MRL-*Mir223*$^{-/-}$*Fas*$^{lpr/lpr}$ (n=4) and MRL-*Mir223*$^{+/+}$*Fas*$^{lpr/lpr}$ (n=4) mice. (**C**) Western blot analyses of Bcl-xL, Bcl-2 and Caspase-3 in axillary lymph nodes and spleen MRL-*Mir223*$^{-/-}$*Fas*$^{lpr/lpr}$ (n=3) and MRL-*Mir223*$^{+/+}$*Fas*$^{lpr/lpr}$ (n=3) mice. Band intensity (normalized to total β -actin) measured by using ImageJ software. Quantification of Western blot results (right side). (n=3 per group). Data are presented as mean ± SEM. *$p < 0.05$, ns = not significant, by Student's t-test in (**A**) and (**B**), by Wilcoxon signed-rank test in (**C**).

Supplementary Figure 6. The *S1pr1* and miR-223-3p expression in B6-*Mir223*$^{-/-}$*Fas*$^{lpr/lpr}$mice. (**A)** mRNA expression of *S1pr1* in $CD4^+$ T cells, $CD8^+$ T cells and B cells isolated from whole cervical lymph nodes and spleen in B6-*Mir223*$^{-/-}$*Fas*$^{lpr/lpr}$ (n=3) and B6-*Mir223*$^{+/+}$*Fas*$^{lpr/lpr}$ (n=3) mice. Data are presented as mean ± SEM. *$p < 0.05$, ns = not significant, by Student's t-test. (**B)** The expression level of miR-223-3p in $CD4^+$ T

cells, $CD8^+$ T cells and B cells isolated from whole cervical lymph nodes and spleen in B6-*Mir223*$^{-/-}$*Fas*$^{lpr/lpr}$ (n=3) and B6-*Mir223*$^{+/+}$*Fas*$^{lpr/lpr}$ (n=3) mice. (**C**) Western blot analyses of S1PR1 in $CD4^+$ T cell from cervical lymph nodes and spleen of B6-*Mir223*$^{-/-}$*Fas*$^{lpr/lpr}$ (n=3) and B6-*Mir223*$^{+/+}$*Fas*$^{lpr/lpr}$ (n=3) mice. Band intensity (normalized to total β -actin) measured by using ImageJ software. Quantification of Western blot results (right side). (n=3 per group). Data are presented as mean ± SEM. **p* < 0.05, by Wilcoxon signed-rank test.

Supplementary Figure 7. Exacerbation of lupus nephritis in B6-*Mir223*$^{-/-}$*Fas*$^{lpr/lpr}$ mice. (**A)** Deposition of C3 and IgG as evaluated by direct immunofluorescence, in the kidneys from B6-*Mir223*$^{-/-}$*Fas*$^{lpr/lpr}$ and B6-*Mir223*$^{+/+}$*Fas*$^{lpr/lpr}$ mice. Images are representative of 5 mice per group. Bars=50 μm. (**B)** Immunofluorescence intensity of C3 and IgG deposits, as evaluated using computed image analysis software. The final fluorescence intensity score reflects the average of at least 5 glomeruli per mouse. (**C)** Glomerular area, mesangial matrix area and numbers of glomerular nuclei in B6-*Mir223*$^{-/-}$*Fas*$^{lpr/lpr}$ compared to B6-*Mir223*$^{+/+}$*Fas*$^{lpr/lpr}$ mice (n=10 per group). Data are presented as mean ± SEM. **p* < 0.05, ***p* < 0.01, ****p* < 0.001, ns = not significant, by Wilcoxon signed-rank test in (**B)**, by Student's t-test in scores in (**C)**.

Supplementary Figure 8. Glomerulonephritis with infiltration of $CD4^+S1PR1^+$ T cells in in B6-*Mir223*$^{-/-}$*Fas*$^{lpr/lpr}$ (**A)** Levels of proteinuria in B6-*Mir223*$^{-/-}$*Fas*$^{lpr/lpr}$ (n=10) and B6-*Mir223*$^{+/+}$*Fas*$^{lpr/lpr}$ (n=11) mice. (**B)** Organ weights at 44 weeks in B6-*Mir223*$^{-/-}$*Fas*$^{lpr/lpr}$ (n=16) and B6-*Mir223*$^{+/+}$*Fas*$^{lpr/lpr}$ (n=17) mice. (**C)** The image of $CD4^+$ cells,

$CD4^+S1PR1^+$ cells, $CD8^+$ cells and $CD8^+S1PR1^+$ cells in tubular lesion in B6-*Mir223*$^{-/-}$*Fas*$^{lpr/lpr}$ compared to B6-*Mir223*$^{+/+}$*Fas*$^{lpr/lpr}$ mice (n = 5 per group). (**D**) mRNA expression of *Cxcl9, Cxcl10, Cxcl11, Ccl2, Ccl4* and *Ccl5* in renal cortex from B6-*Mir223*$^{-/-}$*Fas*$^{lpr/lpr}$ (n=5) and B6-*Mir223*$^{+/+}$*Fas*$^{lpr/lpr}$ (n=5) mice. Images are representative of 5 mice per group. Bars=50 μm. Data are presented as mean ± SEM. *$p < 0.05$, **$p < 0.01$, ***$p < 0.001$, ns = not significant, by Wilcoxon signed-rank test in (**A)** and (**D**), by Student's t-test in (**B)**.

Supplementary Figure 9. Exacerbation of lupus nephritis in *Mir223*$^{-/-}$*Fas*$^{lpr/lpr}$ mice. (**A)** Deposition of C3 and IgG as evaluated by direct immunofluorescence, in the kidneys from MRL-*Mir223*$^{-/-}$*Fas*$^{lpr/lpr}$ and MRL-*Mir223*$^{+/+}$*Fas*$^{lpr/lpr}$ mice. Images are representative of 5 mice per group. Bars=50 μm. Representative kidney sections from MRL-*Mir223*$^{-/-}$*Fas*$^{lpr/lpr}$ and MRL-*Mir223*$^{+/+}$*Fas*$^{lpr/lpr}$ mice, stained with periodic acid–Schiff (PAS) for analysis of glomerular size and mesangial expansion. Bars=50 μm. (**B)** Immunofluorescence intensity of C3 and IgG deposits, as evaluated using computed image analysis software. The final fluorescence intensity score reflects the average of at least 5 glomeruli per mouse. (**C**) Score distribution of glomerular, renal vascular and tubulointerstitial lesions in MRL-*Mir223*$^{-/-}$*Fas*$^{lpr/lpr}$ compared to MRL-*Mir223*$^{+/+}$*Fas*$^{lpr/lpr}$ mice (n=10 per group). Glomerular area, mesangial matrix area and numbers of glomerular nuclei in MRL-*Mir223*$^{-/-}$*Fas*$^{lpr/lpr}$ compared to MRL-*Mir223*$^{+/+}$*Fas*$^{lpr/lpr}$ mice (n=10 per group). Data are presented as mean ± SEM. *$p < 0.05$, **$p < 0.01$, ***$p < 0.001$, ns = not significant, by Wilcoxon signed-rank test in (**B**), by Pearson's $\chi 2$ test and by Student's t-test in scores in (**C)**.

Supplementary Figure 10. Glomerulonephritis with infiltration of $CD4^{+}$ T cells in *Mir223*$^{-/-}$*Fas*$^{lpr/lpr}$ mice. (**A)** Levels of proteinuria in MRL-*Mir223*$^{-/-}$*Fas*$^{lpr/lpr}$ (n=10) and MRL-*Mir223*$^{+/+}$*Fas*$^{lpr/lpr}$ (n=11) mice. (**B)** The number of $CD4^{+}$ cells, $CD4^{+}S1PR1^{+}$ cells, $CD8^{+}$ cells and $CD8^{+}S1PR1^{+}$ cells in glomerulus or tubular lesion in MRL-*Mir223*$^{-/-}$ *Fas*$^{lpr/lpr}$ compared to MRL-*Mir223*$^{+/+}$*Fas*$^{lpr/lpr}$ mice (n = 5 per group). (**C)** The image of $CD4^{+}$ cells, $CD4^{+}S1PR1^{+}$ cells, $CD8^{+}$ cells and $CD8^{+}S1PR1^{+}$ cells in glomerular and tubular lesion in MRL-*Mir223*$^{-/-}$*Fas*$^{lpr/lpr}$ compared to MRL-*Mir223*$^{+/+}$*Fas*$^{lpr/lpr}$ mice (n = 5 per group). Images are representative of 5 mice per group. Bars=50 μm. Data are presented as mean ± SEM. *$p < 0.05$, **$p < 0.01$, ***$p < 0.001$, ns = not significant, by Student's t-test in (**A) and (B).**

Supplementary Figure 11. mRNA expression of *Cxcl9, Cxcl10, Cxcl11, Ccl2, Ccl4* and *Ccl5* in renal cortex from MRL-*Mir223*$^{-/-}$*Fas*$^{lpr/lpr}$ (n=5) and MRL-*Mir223*$^{+/+}$*Fas*$^{lpr/lpr}$ (n=5) mice. Data are presented as mean ± SEM. ns = not significant, by Wilcoxon signed-rank test.

Supplementary Figure 1

A

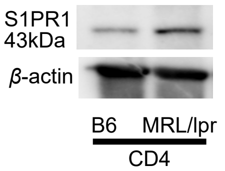

B

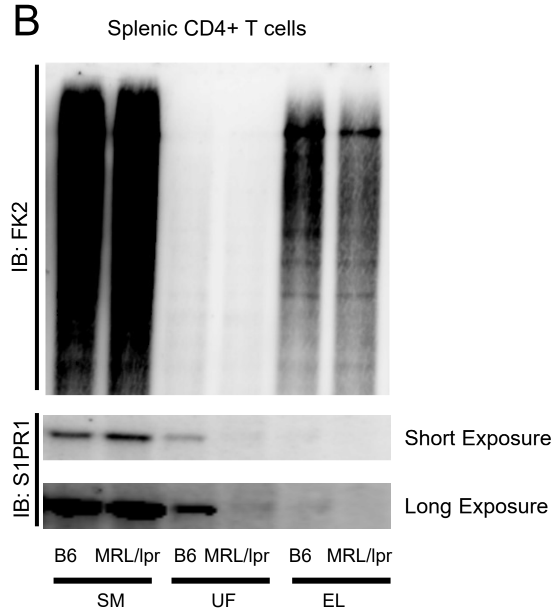

Supplementary Figure 2

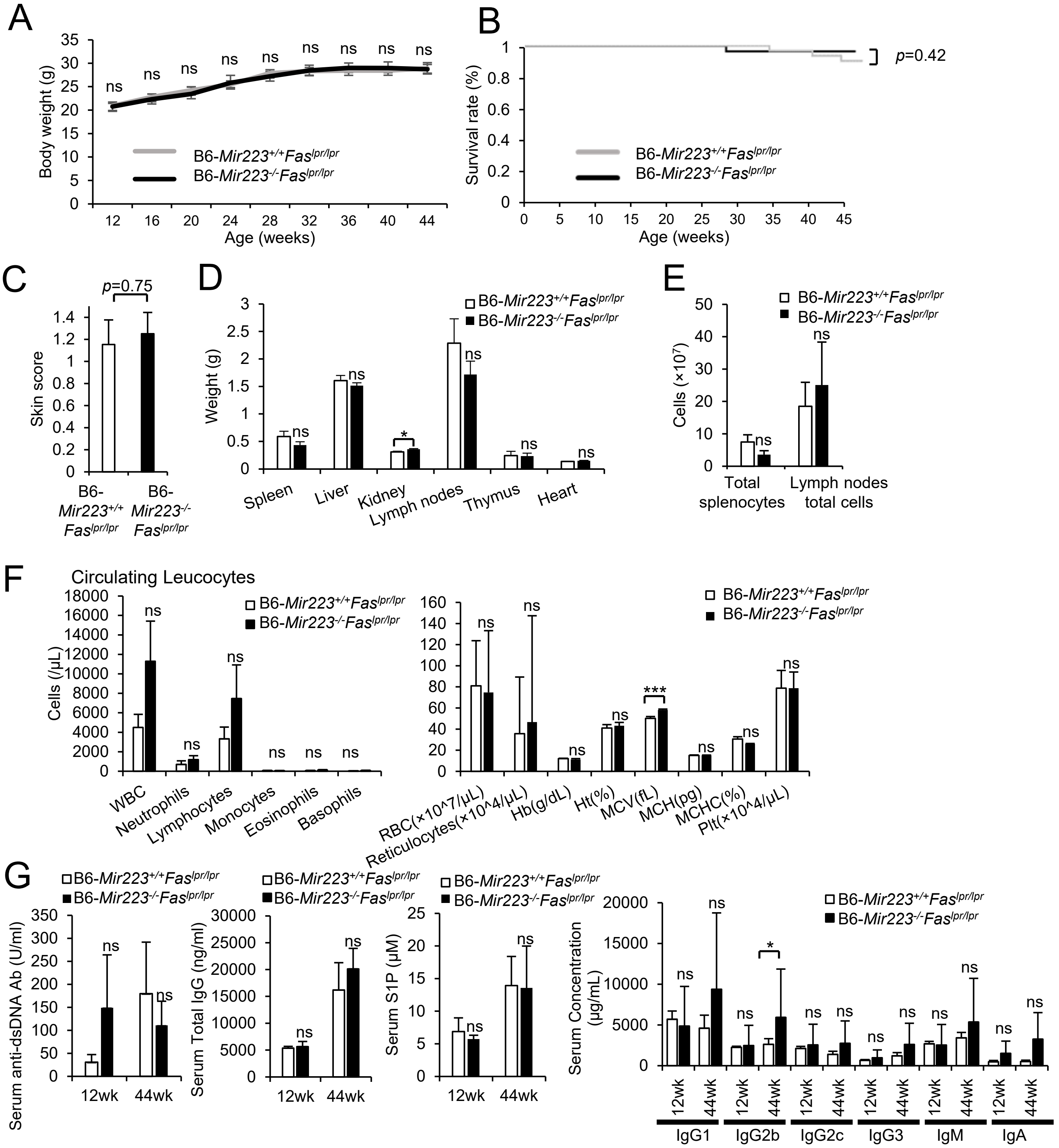

Supplementary Figure 3

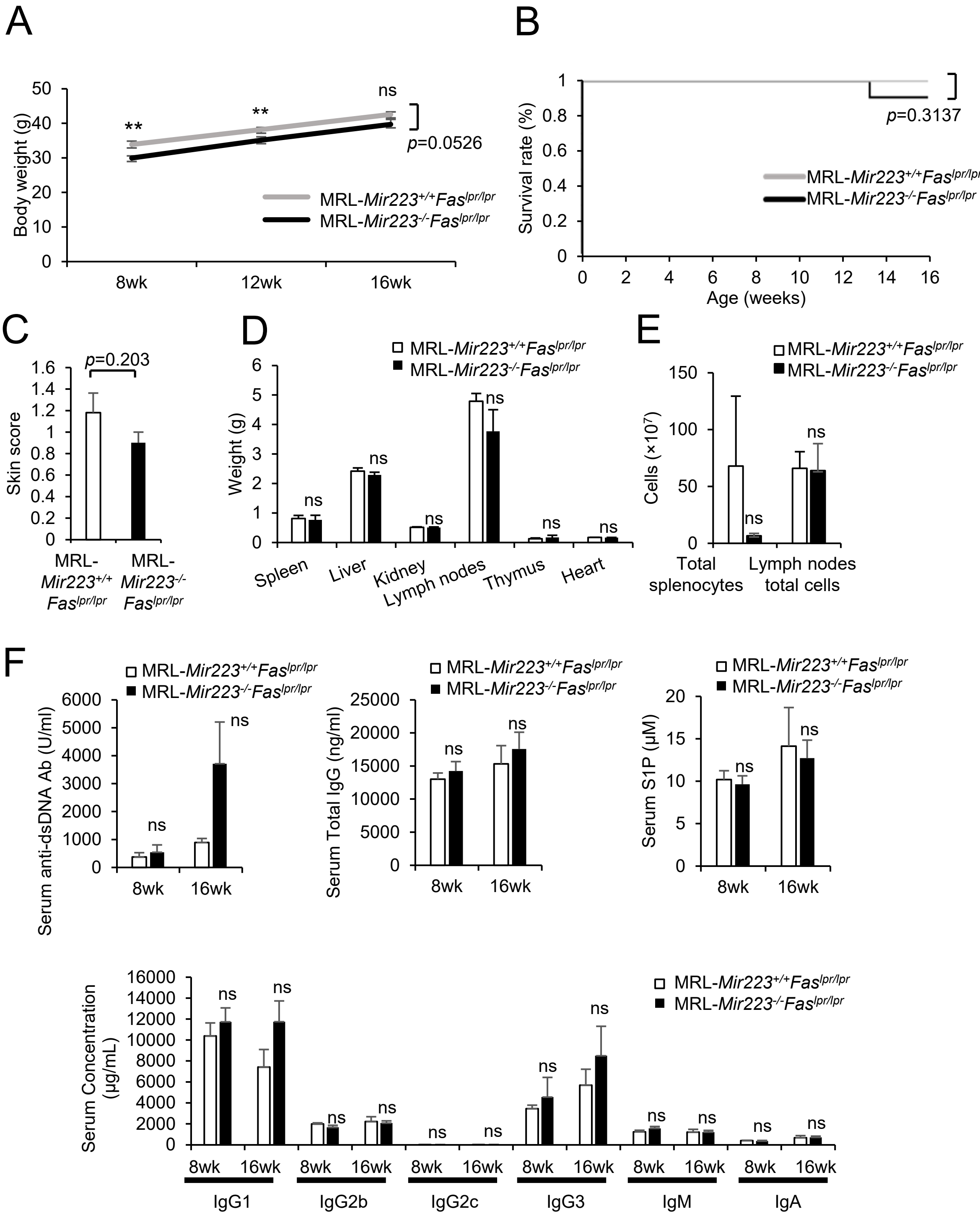

Supplementary Figure 4

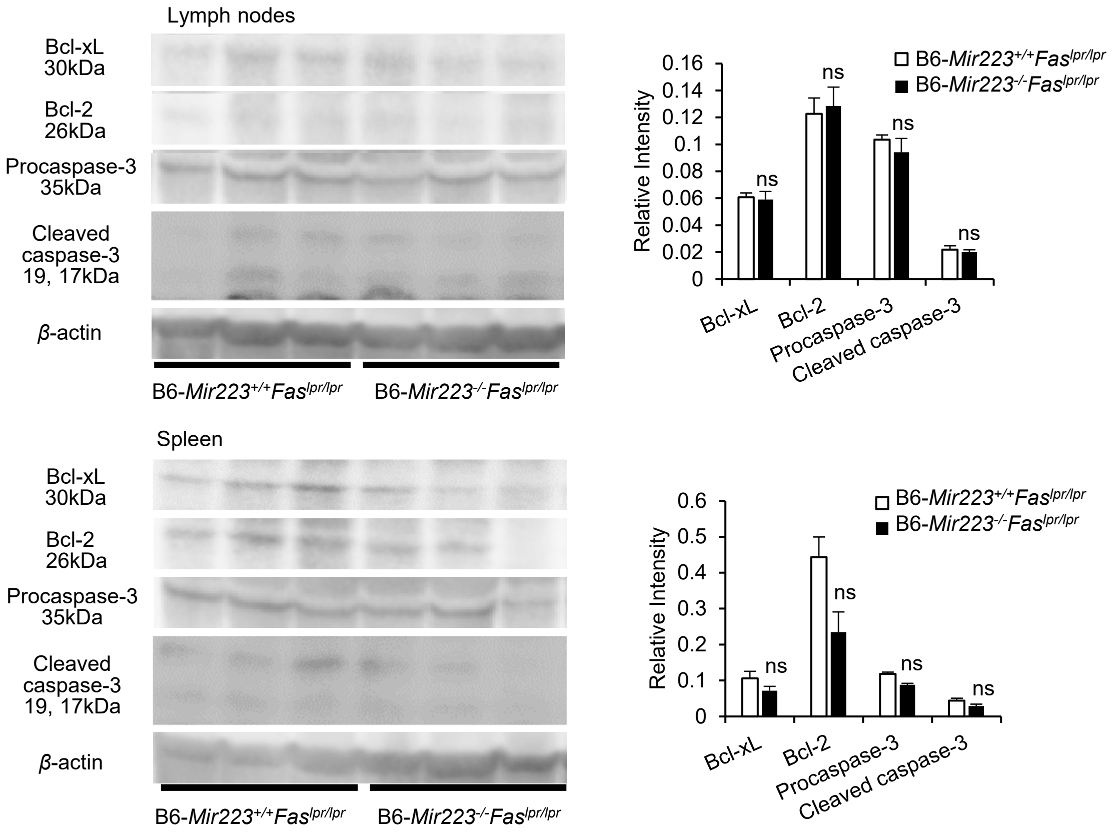

Supplementary Figure 5

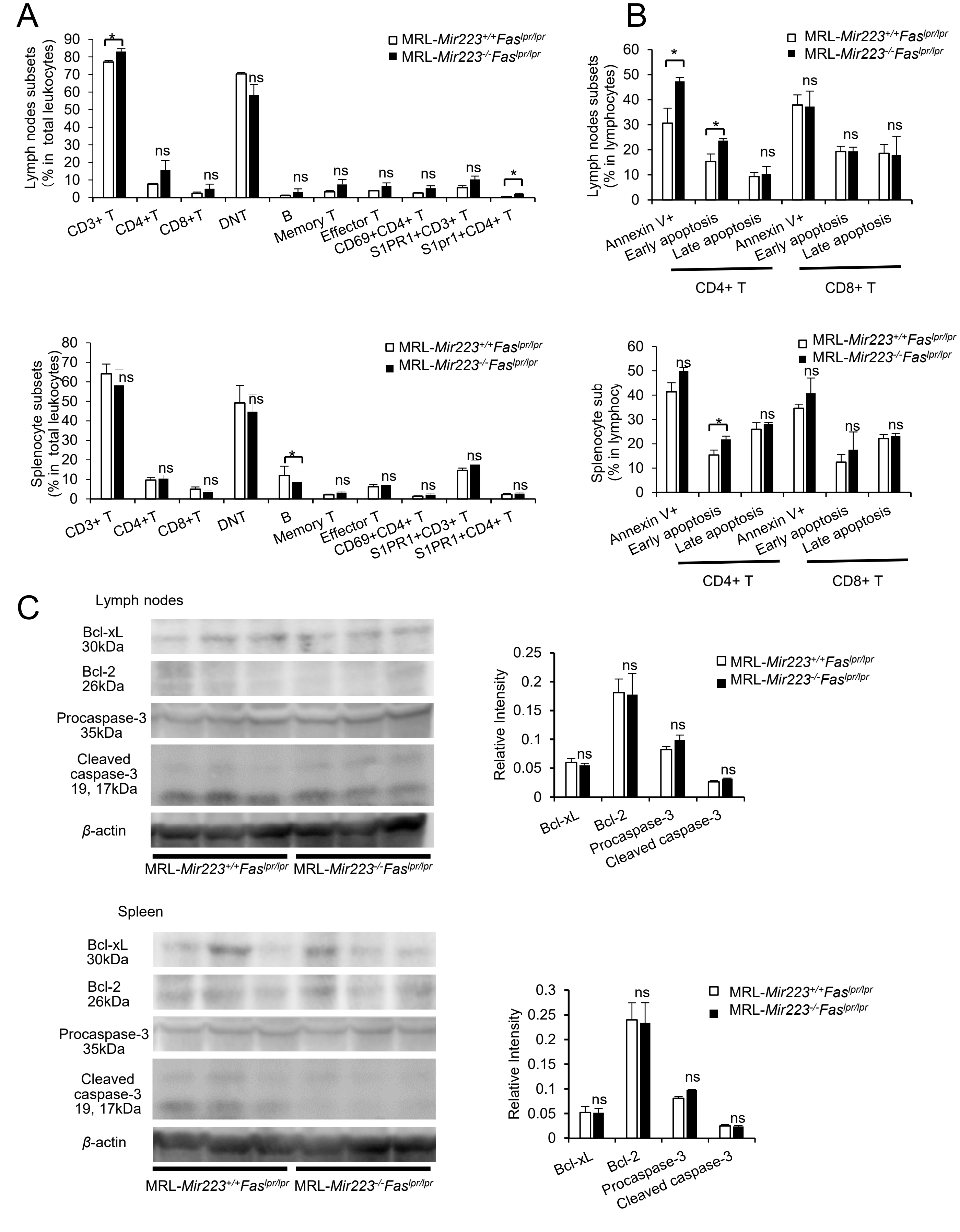

Supplementary Figure 6

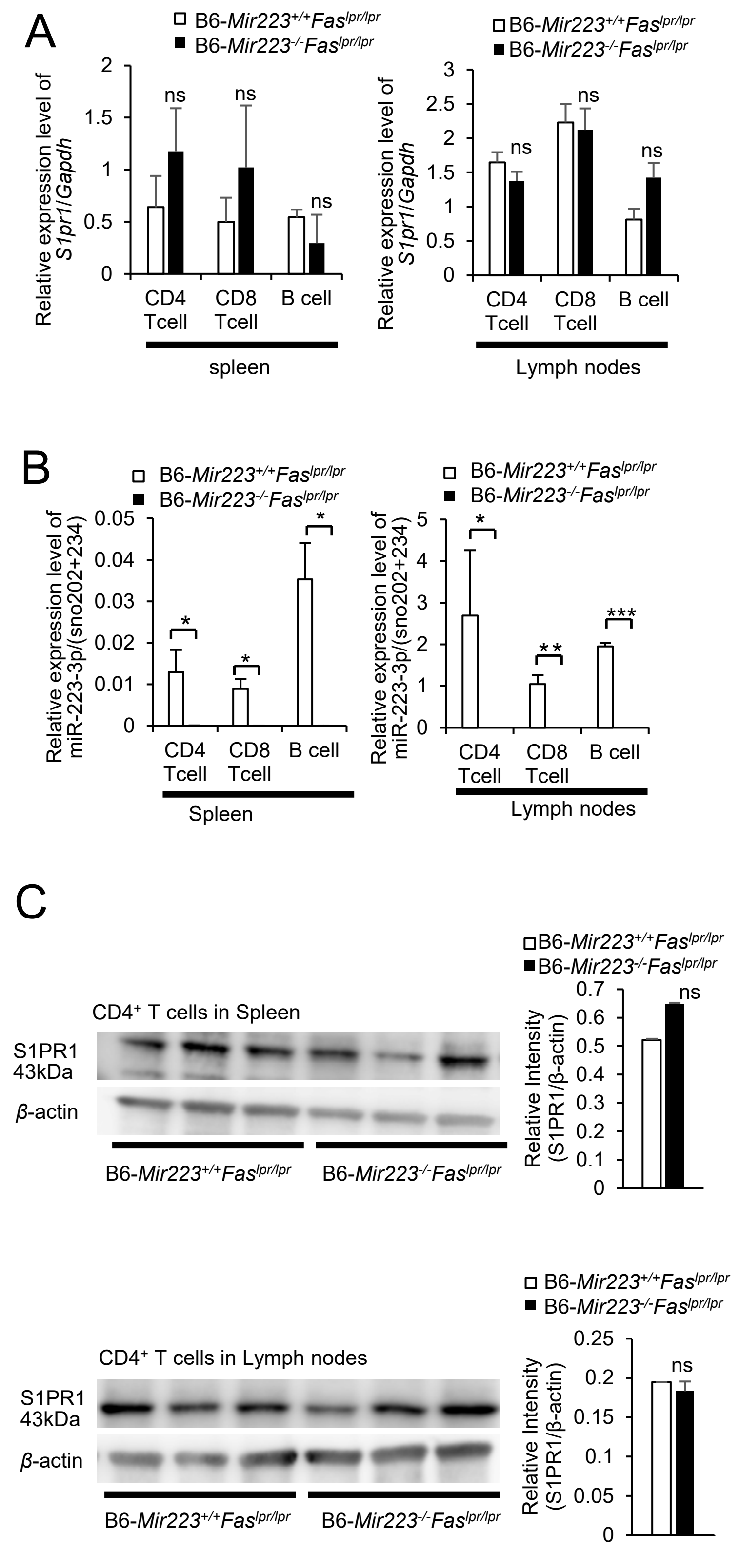

Supplementary Figure 7

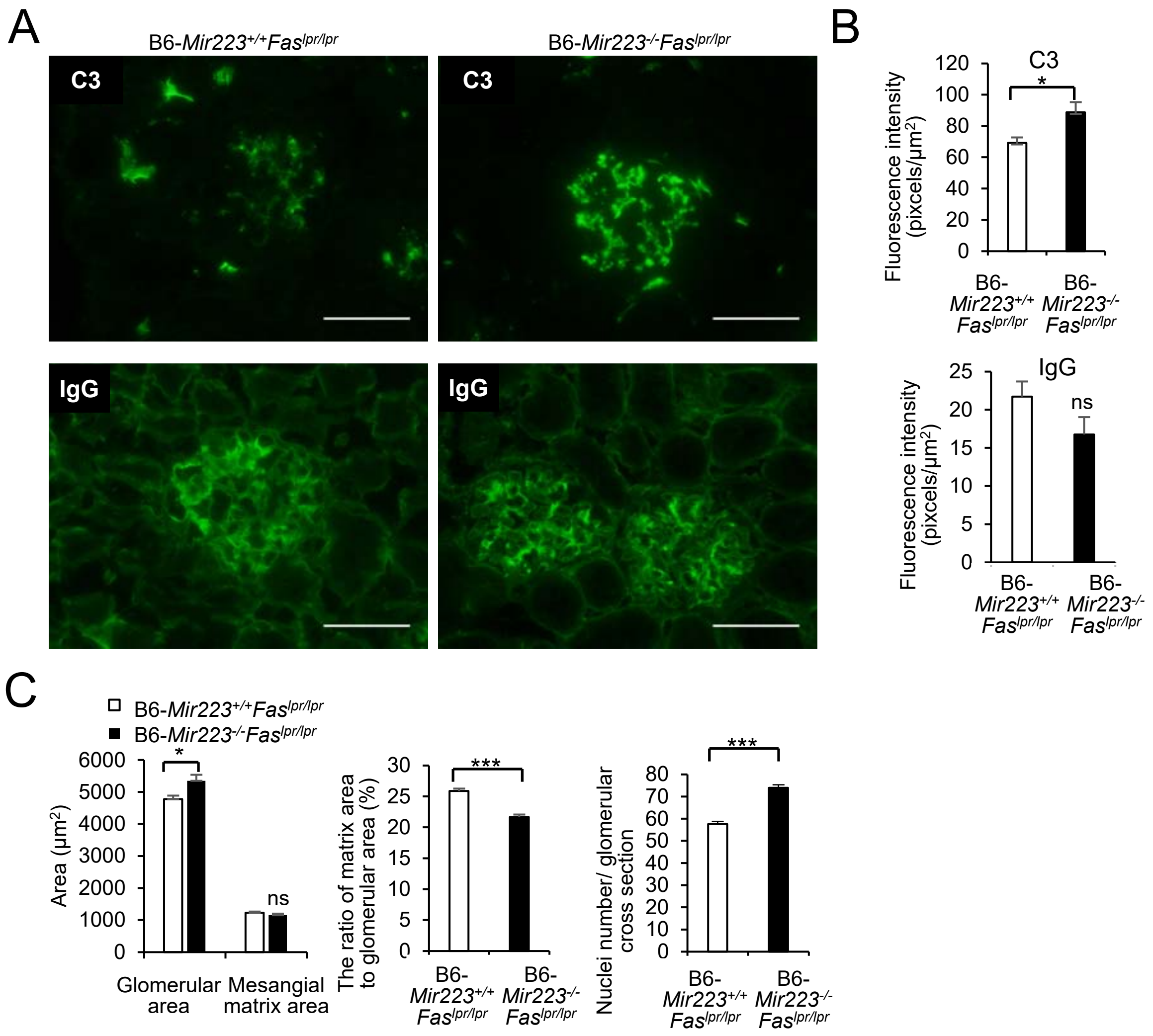

Supplementary Figure 8

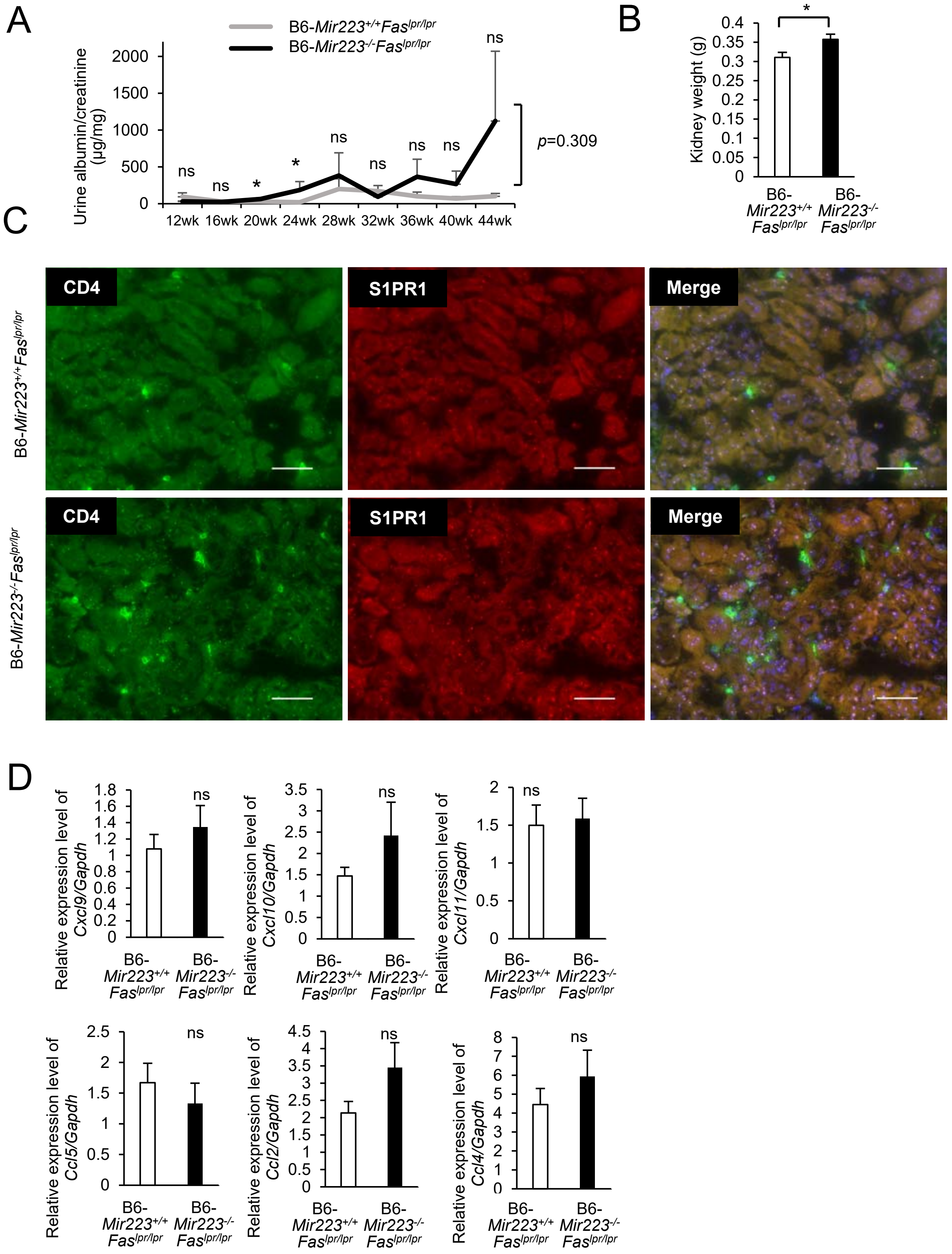

Supplementary Figure 9

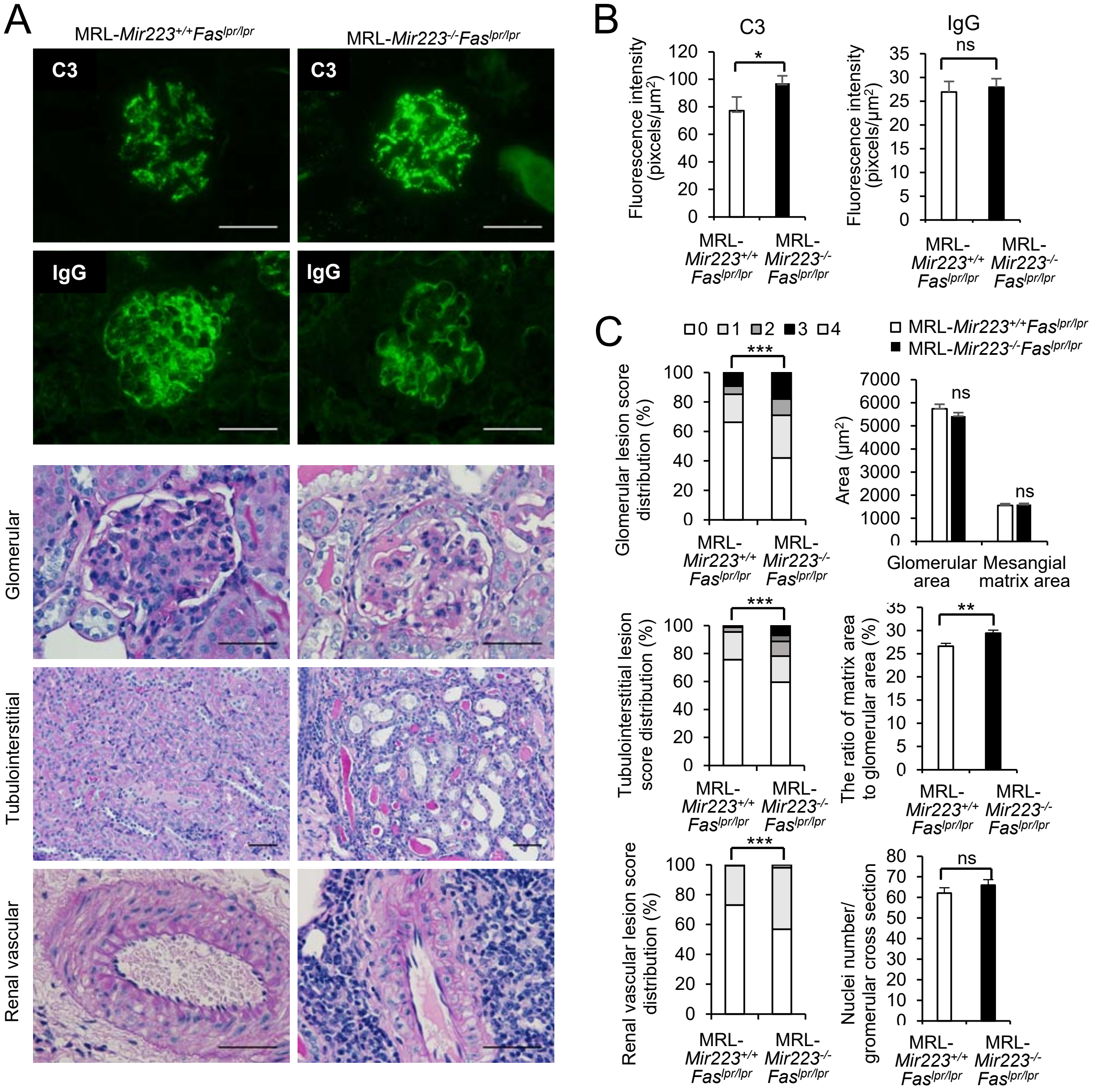

Supplementary Figure 10

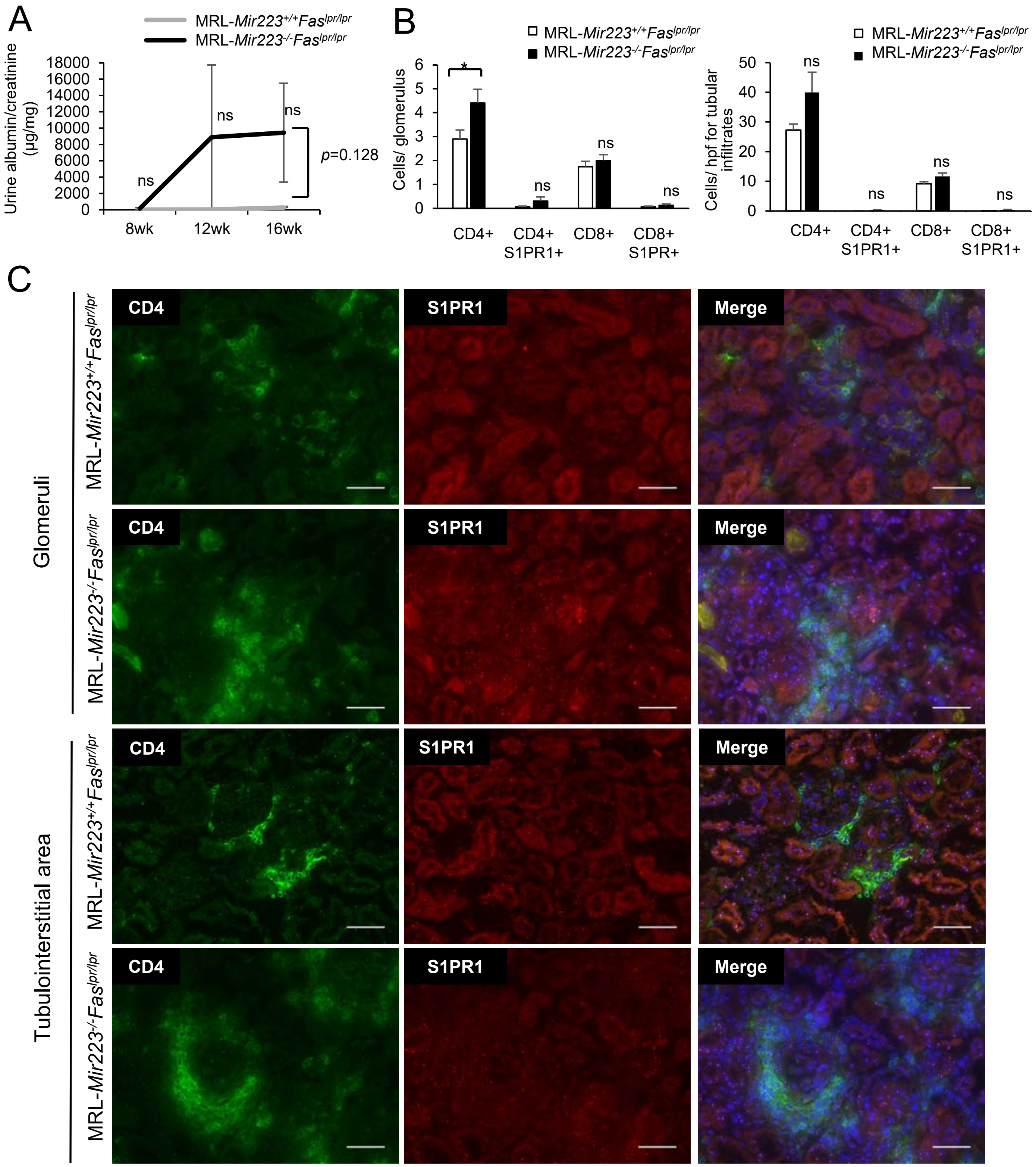

Supplementary Figure 11

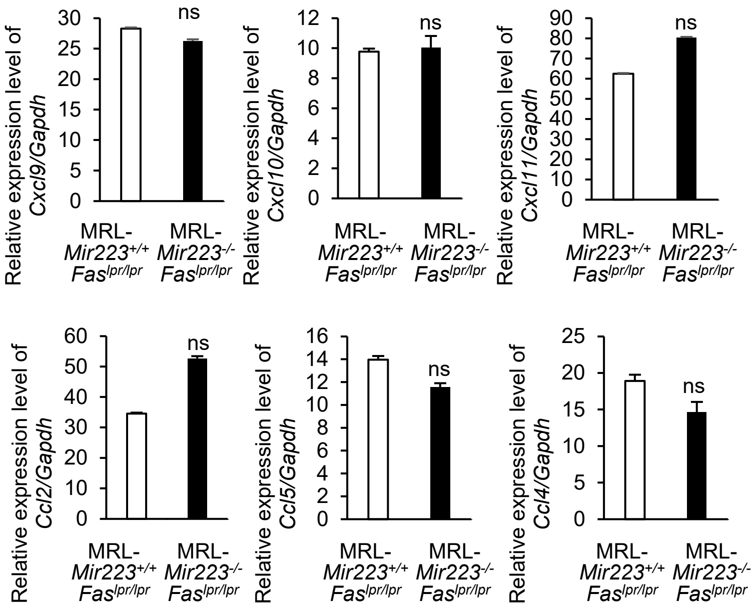

Supplement: Supplementary file 1 [file Image_1.pdf]
